# Tyrosine kinase c-Abl couples RNA polymerase II transcription to DNA double-strand breaks

**DOI:** 10.1093/nar/gkz024

**Published:** 2019-01-22

**Authors:** Kaspar Burger, Margarita Schlackow, Monika Gullerova

**Affiliations:** Sir William Dunn School of Pathology, University of Oxford, South Parks Road, Oxford OX1 3RE, UK

## Abstract

DNA is constantly exposed to endogenous and exogenous damage. Various types of DNA repair counteract highly toxic DNA double-strand breaks (DSBs) to maintain genome stability. Recent findings suggest that the human DNA damage response (DDR) utilizes small RNA species, which are produced as long non-coding (nc)RNA precursors and promote recognition of DSBs. However, regulatory principles that control production of such transcripts remain largely elusive. Here we show that the Abelson tyrosine kinase c-Abl/ABL1 causes formation of RNA polymerase II (RNAPII) foci, predominantly phosphorylated at carboxy-terminal domain (CTD) residue Tyr1, at DSBs. CTD Tyr1-phosphorylated RNAPII (CTD Y1P) synthetizes strand-specific, damage-responsive transcripts (DARTs), which trigger formation of double-stranded (ds)RNA intermediates via DNA–RNA hybrid intermediates to promote recruitment of p53-binding protein 1 (53BP1) and Mediator of DNA damage checkpoint 1 (MDC1) to endogenous DSBs. Interference with transcription, c-Abl activity, DNA–RNA hybrid formation or dsRNA processing impairs CTD Y1P foci formation, attenuates DART synthesis and delays recruitment of DDR factors and DSB signalling. Collectively, our data provide novel insight in RNA-dependent DDR by coupling DSB-induced c-Abl activity on RNAPII to generate DARTs for consequent DSB recognition.

## INTRODUCTION

Transcription of is a fundamental and highly regulated process. The largest subunit of RNAPII contains a low complexity C-terminal domain (CTD), which comprises 52 consensus heptads (Tyr1-Ser2-Pro3-Thr4-Ser5-Pro6-Ser7) and undergoes dynamic, regulatory post-translational modifications (1,2). Phosphorylated CTD residues S2/5P are hallmarks of active transcription of protein-coding genes (3). Y1 phosphorylation is less characterized. In *Saccharomyces cerevisiae*, Y1P blocks the recruitment of termination factors to elongating RNAPII (4). In mammals, Y1P is enriched at promoters and enhancers and is associated with antisense transcription and termination (5–7). Interestingly, Y1P levels are elevated in response to DNA damage by the atypical tyrosine kinase Mpk1/Slt2 in budding yeast and human c-Abl (8,9). c-Abl directly phosphorylates Y1 *in vitro* (4), suggesting a potential link between c-Abl, CTD Y1P and the DDR.

Accurate DDR is essential for genome stability (10). Unscheduled, excessive RNA synthesis may threat the genome as it implicates elevated exposure of unprotected DNA (11). Thus, transcription is globally impaired in response to DSBs by physical blockage and degradation of RNAPII (12–14), concomitant with formation of non-permissive heterochromatin and silencing of transcribed lesions (15,16). Intriguingly, the chromatin state impacts on genome stability, with heterochromatic regions driving mutation rates (17). DSBs are repaired faster, if they occur at actively transcribed loci (18). Data utilizing the sequence-specific *Asi*SI-ER endonuclease demonstrate that acetylated histone marks associated with active transcription accumulate at a subset of DSBs, and that RNAPII occupancy correlates with nucleosome-free regions rather than being disengaged from *Asi*SI-ER-restricted sites (19). Thus, DSBs may trigger chromatin breathing to create a local and transiently permissive window of opportunity for RNAPII localization and activity (20). Indeed, increasing evidence suggests that DSBs can promote gene expression locally and engage small ncRNA for repair (21,22). Site-specific DNA damage response RNA (DDRNA) accumulate at DSBs in various organisms (23–26), which involves production of strand-specific, long non-coding precursors (27). Such transcripts may originate from genic and intergenic DSBs and undergo processing by RNA interference (RNAi) factors Drosha and Dicer. DDRNA facilitate recruitment of secondary DDR factors 53BP1 and MDC1 to establish DSB foci, but not primary DDR factors, such as the Mre11–Rad50–Nbs1 (MRN) complex (28). We have recently shown that nuclear phosphorylated Dicer (p-Dicer) accumulates at DSBs upon phosphatidylinositol-3-kinase related kinase (PIKK)-dependent phosphorylation to processes endogenous, DSB-induced dsRNA and promote recruitment of 53BP1 and MDC1 to DSBs in mammals (29,30). In search for p-Dicer substrates, we here show c-Abl-dependent formation of CTD Y1P foci at DSBs. CTD Y1P produces strand-specific, damage-responsive transcripts (DARTs), which lead to formation of dsRNA and are subject to p-Dicer processing to amplify RNA-dependent recruitment of 53BP1 and MDC1 to DSBs.

## MATERIALS AND METHODS

### Tissue culture and transfections

Human wild type or *Asi*SI-ER expressing U2OS, HEK293, HeLa, MCF7, SKBR3 and murine MEF cells were cultured in DMEM (Gibco), supplemented with 10% non-stripped fetal bovine serum (Life Tech.), 100 U/ml Penicillin, 100 μg/ml Streptomycin and 2 mM L-glutamine (Gibco) at 37°C and 5% CO_2_. Transient transfection of plasmids encoding *Asi*SI-ER (pBABE::*Asi*SI-ER, gift from Fabrizio d’Adda di Fagagna), full length, HA-tagged 53BP1-encoding plasmid pHAGE*-*N*-*FLAG*-*HA-53BP1 (gift from Ross Chapman), GFP-tagged RNaseH1-encoding pEGFP-M27 plasmid (gift from Nadina Skourti-Stathaki), Copepoda (Cop)GFP (pmax-GFP vector, Lonza), or enhanced GFP-tagged Abl1 kinase active (eGFP-Abl-KA) and kinase dead (eGFP-Abl-KD) variants (gifts from Han Seok Ko) or small-interfering (si)RNA (100 nM) was performed using Lipofectamine 2000 (Invitrogen) and Opti-MEM (Invitrogen). For RNAi knockdown, cells were incubated for 6 h with transfection reagents on two consecutive days.

### RNA interference (RNAi)

siRNA sequences (5′-3′) were: siControl (ON-TARGETplus, Dharmacon SMARTpool, #D-001810-01-05, Dharmacon, scrambled sequence); siMre11, ACAGGAGAAGAGAUCAACUdTdT (Sigma); siATM (ON-TARGETplus, Dharmacon SMARTpool, human ATM, #L-003201-00-0005); siATR (ON-TARGETplus, Dharmacon SMARTpool, human ATR, #L-003202-00-0005); siDNA-PKcs (ON-TARGETplus, Dharmacon SMARTpool, human PRKDC, #L-005030-00-0005); siAbl (ON-TARGETplus, Dharmacon SMARTpool, human Abl1, #L-003100-00-0005); siAbl-UTR AUCAACAAACUGGAGAAUAdTdT (Sigma). Short-hairpin (sh)RNA-encoding plasmids were used for depletion of Dicer (Mission shDicer NM_030621; Sigma, #10271413MN) or scrambled depletion (Mission shControl pLKO.1-puro non-target shRNA, Sigma, #SHC016).

### Antibodies and small molecule inhibitors

Primary antibodies were: anti-α-Tubulin (Abcam, [YL1/2], ab6160); anti-β-Tubulin (Abcam, ab6046); anti-γH2A.X (S139, Merck Millipore, 05-636); anti-eGFP (GeneTex, [GT859], GTX628528); anti-eGFP (Abcam, ab290); anti-Rad21 (Merck Millipore, 05-908); anti-ATM (Santa Cruz, [2C7], sc-23921); anti-phospho-ATM (S1981, Abcam, ab81292); anti-53BP1 (Santa Cruz, [H-300], sc-22760); anti-histone H3 (Abcam, ab1791); anti-RNAPII N20X (Santa Cruz, sc-899 X); anti-RNAPII 8WG16 (Abcam, ab817); anti-RNAPII-CTD phospho-Ser2 (Abcam, ab5095); anti-RNAPII-CTD phospho-Ser5 (Abcam, ab5131); anti-RNAPII-CTD phospho-Tyr1 (Active Motif, [3D12], 61383); anti-Nbs1 (Sigma, N3162); anti-Mre11 (GeneTex, [12D7], GTX70212); anti-DNA–RNA hybrid (Kerafast, [S9.6], ENH001); anti-DNA–RNA hybrid (Merck-Milipore, [S9.6], MABE1095); anti-single-stranded DNA (Merck-Milipore, [16-19], MAB3034); anti-c-Abl/Abl1 (Abcam, ab15130); anti-c-Abl/Abl1 (CST, 2862); anti-phospho-c-Abl/p-c-Abl1 (Y245, Thermo, 44-250); anti-phospho-c-Abl/p-c-Abl1 (Y245, Abcam, ab62189); anti-MDC1 (GeneTex, [N2N3], GTX102673); anti-ATR (GeneTex, [2B5], GTX70109); anti-DNA-PKcs, catalytic subunit (cs) (Abcam, [18-2], ab1832); anti-HA-tag (Roche, [3F10], 000000011867423001); anti-Ki-67 (Abcam, [SP6], ab16667); anti-Dicer (A-2, Santa Cruz, sc-136891); anti-J2 (Scicons, 10010200); anti-p53 (DO-I, MA5-12571, Thermo); anti-phospho-Chk1 (S317, CST); anti-p-DCR-1 (Ser1712/Ser1836, a kind gift from the Arur Lab (31). p-DCR-1 signals represent a mixture of two individual antibodies, raised against carboxy-terminal murine Dicer epitopes phospho-Ser1712 and phospho-Ser1836 individually in separate rabbits.

Cells were incubated with ATM inhibitor KU-55933 (5 μM, Sigma), ATR inhibitor VE-821 (1 μM, Sigma), PIKK inhibitor LY294002 (5 μM, NEB), c-Abl inhibitors Imatinib (1 μM) or Ponatinib (1 μM) (both gifts from Kilian Huber), or 4-thiouridine (100 μM, Sigma) for 1 h, Flavopiridol (500 nM, Sigma), THZ1 (1 μM, Merck), Leptomycin B (5 nM, Cayman) or Mirin (100 μM, Cayman) for 2 h, or α-Amanitin (2 μg/ml, Sigma) for 24 h prior to induction of DSBs by 4-hydroxytamoxifen (300 nM, Cayman) for 4 h. For γ-irradiation a total dose of 10 Gy was used and cells were harvested 1 h post irradiation, unless stated differently.

### Cell sorting

For fluorescence-activated cell sorting (FACS), cells were fixed in cold ethanol and permeabilized in PBS/0.1% Triton X-100 in presence of recombinant RNaseA (0.2 mg/ml, Sigma). Nuclei were stained in 1 mg/ml propidium iodide (Sigma), counted in FL2/FL3 channels on a FACSCalibur flow cytometer (BD Biosciences) and analysed with FlowJo (Tree Star). Doublet cells were excluded by measuring peak area and width. For analysis of GFP-RNaseH1 overexpression, cells were transfected with pEGFP-M27 and equal numbers of GFP-positive and -negative cells were sorted by flow cytometry 24 h prior to analysis.

### Protein analytics and immunoprecipitation

Protein levels were assessed as whole cell extracts, directly lysed, boiled and sonicated in 4× SDS Laemmli buffer (250 mM Tris–HCl pH 6.8, 8% SDS, 40% glycerol, 8% β-mercaptoethanol, 0.02% bromophenol blue). Samples were separated by SDS-PAGE using precast gels (Mini-PROTEAN TGX, BioRad), transferred onto nitrocellulose membranes (Protran, GE Healthcare) and probed with antibodies.

For immunoprecipitation, cells were trypsinized, washed in cold 1× PBS and centrifuged (1200rpm, 5 min). Pellets were lysed in 5 volumes lysis buffer (100 mM KCl, 5 mM MgCl_2_, 10 mM HEPES pH 7.0, 0.5% NP-40, 1 mM DTT, 100 U RNase inhibitor, 1× protease/phosphatase inhibitor cocktails, Roche) for 20 min on ice. Lysates were centrifuged (12 000 rpm, 12 min) and supernatants were incubated with 5 ug primary antibodies, which were coupled to magnetic beads according to the manufacturer's protocol (Invitrogen) or beads only for 2 h at 4°C, pulled down, washed 3× for 10 min with 800 μl lysis buffer at 4°C and eluted with SDS-PAGE sample buffer by boiling (5 min, 95°C).

### Chromatin immunoprecipitation (ChIP)

Cells were fixed with 1% formaldehyde (10 min, 37°C). Formaldehyde was inactivated by addition of glycine to a final concentration of 0.125 M (10 min at 37°C). Cells were washed 2× with 5 ml cold PBS and centrifuged (5 min, 1600 rpm). Pellets were resuspended in 500 μl cell lysis buffer (5 mM PIPES pH 8.0, 85 mM KCl, 0.5% NP-40, 1× protease/phosphatase inhibitor cocktails, Roche) and incubated (10 min on ice). Nuclei were collected by centrifugation (5 min, 3000 rpm, 4°C) and resuspended in 400 μl cold nuclear lysis buffer (1% SDS, 10 mM EDTA, 50 mM Tris–HCl pH 8.0, 1× protease/phosphatase inhibitor cocktails, Roche) and incubated (10 min on ice). Samples were sonicated to an average length of 300–500 bp, kept on ice (5 × 30 s on/off for 5 min each) and spun (10 min, 13 000 rpm, 4°C). The supernatant was diluted with 2.5 volumes immunoprecipitation dilution buffer (0.01% SDS, 1.1% Triton X-100, 1.2 mM EDTA, 16.7 mM Tris–HCl pH 8.0, 167 mM NaCl, 1× protease/phosphatase inhibitor cocktails, Roche). Diluted ChIP samples were precleared by incubation with protein A/G agarose beads (Merck-Millipore) or magnetic beads (Invitrogen) for 30 min and aliquoted into various immunoprecipitation samples. Antibodies (5 μg/100 μg chromatin) were added to samples and incubated (4°C, overnight with rotation). Immune complexes were pulled down at 4°C with 40 μl of protein A/G agarose beads or magnetic beads for 1 h and washed with buffers A–D: A, 0.1% SDS, 1% Triton X-100, 2 mM EDTA, 20 mM Tris–HCl pH 8.0 and 150 mM NaCl; B, 0.1% SDS, 1% Triton X-100, 2 mM EDTA, 20 mM Tris–HCl pH 8.0 and 500 mM NaCl; C, 0.25 M LiCl, 1% NP-40, 1% sodium deoxycholate, 1 mM EDTA and 10 mM Tris–HCl pH 8.0; D, 10:1 TE pH 8.0. Immune complexes were eluted with 500 μl immunoprecipitation elution buffer (1% SDS, 0.1 M NaHCO_3_) for 30 min on a rotating wheel. Reversal of cross-links was performed by adding 0.3 M NaCl, 3 μg/ml RNaseA, 10 μl of 0.5 M EDTA, 20 μl of 1 M Tris–HCl pH 6.5 and 2 μl of 10 mg/ml Proteinase K and incubating at 65°C overnight. DNA was purified by phenol/chloroform extraction and recovered in ddH_2_O. Signals represent the average of at least three biological repeats expressed as a percentage of the input signal using qRT-PCR and comparative quantitation.

### Confocal microscopy

Cells were washed in 1× PBS, fixed on coverslips with 3% formaldehyde in PBS for 10 min, washed and incubated with 50 mM ammonium chloride in PBS (10 min), washed in PBS, permeabilized with PBS/0.1% Tween (7 min) and blocked with PBS/10% FBS (2 h, 4°C). Primary antibodies were incubated overnight at 4°C in PBS/0.15% FBS. Alexa Flour 488-, 546-, 555- or 647-conjugated secondary antibodies (Invitrogen) were incubated in PBS/0.15% FBS (2 h, RT) in a light-protected humidified chamber. Nuclei were counterstained and mounted with 6-diamidino-2-phenylindole (DAPI)-containing Mowiol (Merck). Samples were imaged by confocal microscopy (Olympus FV1000) using equal exposure times, at optical thickness 0.5 μm. Data were acquired with MetaMorph software (Molecular Dynamics) and quantified using RGB profiler (ImageJ, NIH). Quantitations represent a number of cells that have shown phenotype or % of positive cells, see figure legends for details.

### RNA analytics

Total RNA was isolated using TRIzol reagent (Invitrogen). Samples were treated with DNase I (1 U, Roche) for 1 h at 37°C, followed by heat inactivation (10 min, 75°C). cDNA was synthetized using SuperScript III Reverse Transcriptase (Invitrogen) and gene-specific primers (Supplementary Table S1). Quantitative real-time PCR (qRT-PCR) was performed as described (32,33). qRT-PCR data are shown as % of input, ratios or fold-change relative to controls. Nascent RNA and mammalian nascent elongating transcripts were purified using 4-thiouridine-(4sU)-tagging or immunoprecipitation of mammalian nascent elongating transcripts (mNET-IP), as described (34,35).

For mNET-IP, 4 μg antibodies were coupled to magnetic beads (Invitrogen) overnight, washed and resuspended in 100 μl NET-2 buffer (50 mM Tris–HCl pH7.4, 150 mM NaCl, 0.05% NP-40) prior to immunoprecipitation. Cells were harvested, washed in cold PBS and lysed in hypotonic lysis buffer (10 mM HEPES pH 7.9, 60 mM KCl, 1.5 mM MgCl_2_, 1 mM EDTA, 1 mM DTT, 0.075% NP-40, 1× protease/phosphatase inhibitor cocktails, Roche) (10 min, 4°C with rotation). Nuclei were collected by centrifugation (2 min, 1000 rpm, 4°C), washed 2× in hypotonic lysis buffer without NP-40 and resuspended in 125 μl cold NUN1 buffer (20 mM Tris–HCl pH 7.9, 75 mM NaCl, 0.5 mM EDTA, 50% glycerol, 1x protease/phosphatase inhibitor cocktails, Roche). 1.2 ml NUN2 buffer (20 mM HEPES-KOH pH 7.6, 300 mM NaCl, 0.2 mM EDTA, 7.5 mM MgCl_2_, 1% NP-40, 1 M urea, 1× protease/phosphatase inhibitor cocktails, Roche) was added and nuclei were incubated on ice (15 min) with sporadic vortexing and centrifuged (10 min, 16000 rpm, 4°C). The non-soluble chromatin pellet was washed in 100 μl 1× MNase buffer (NEB), centrifuged and digested in 100 μl MNase reaction mix (87 μl ddH_2_O, 10 μl 10× MNase buffer, 1 μl 100× BSA, 2 μl MNase (2000 U/μl) for 90 s at 37°C with rotation). 10 μl 100 mM EDTA was added to stop digestion. MNase digests were centrifuged (5 min, 16 000 rpm, 4°C) and the supernatant was diluted with 10 volumes NET-2 buffer. Prior to dilution, 10% of MNase digests were resuspended and boiled in 1/3 volume of 4× SDS Laemmli buffer. Samples were separated by SDS-PAGE using precast gels (Mini-PROTEAN TGX, BioRad), transferred onto nitrocellulose membranes (Protran, GE Healthcare) and probed with antibodies. Conjugated antibodies were added to the diluted supernatants and incubated for 2 h at 4°C with rotation. mNET-IP was performed in absence of Empigen. Samples were centrifuged (5 min, 2000 rpm, 4°C) and pelleted beads were washed in 800 μl NET-2 buffer 7×. Prior to last wash, 10% of mNET-IP samples were separated for RNA end-labeling. Beads were incubated with 10 μl labeling mix (1 μl 10× PNK buffer, 1 μl 1% Tween, 0.5 μl T4 PNK (NEB), 1 μl γ-^32^P-ATP (Perkin Elmer), 6.5 μl ddH_2_O) for 20 min at 37°C with rotation, washed in 800 μl NET-2 buffer and pelleted. End-labeled RNA as well as the remaining 90% non-labeled RNA was recovered using TRIzol, resuspended in 20 μl urea dye (7 M urea, 0.05% xylene cyanol, 0.05% bromophenol blue), incubated at 75°C for 10 min and separated (30 min, 350 V) in 1× TBE buffer (90 mM Tris, 90 mM boric acid, 2 mM EDTA) on a 6% PAGE gel with 7 M urea. End-labeled separated RNA was transferred on Whatman paper using a gel dryer for 2 h, visualized by autoradiography and quantified by ImageJ (NIH). Migration fronts of xylene cyanol and bromophenol blue or end-labeled pBR322 MspI digest (NEB) were used as size marker. Non-labeled, separated RNA was size-selected into a long (>100 nts) and small (<100 nts) fraction by cutting out gel slices according to size markers. Slices were incubated in 400 μl elution buffer (1 M NaOAc, 1 mM EDTA) (2 h, RT with rotation). Samples were centrifuged (2 min, 13 000 rpm) and supernatants containing eluted RNA were loaded on spin-X-columns (Coster) and centrifuged (1 min, 13 000 rpm). Flow-through was precipitated using 1 ml 100% ethanol and 1 μl glycogen (MP Bio), incubated (20 min, RT) and centrifuged (20 min, 13 000 rpm). Pellets were washed in 70% ethanol, air-dried and recovered in ddH_2_O. Pellets containing higher molecular weight RNA were recovered in 20 μl ddH_2_O and subjected to cDNA synthesis using SuperScript III Reverse Transcriptase (Invitrogen) and region-specific primers. Pellets containing small RNA were resuspended in 6 μl ddH_2_O. RNA quality was controlled using a Bioanalyzer (Agilent).

For 4sU-tagging, cells were lysed and total RNA was extracted using TRIzol. 50 μg total RNA was incubated with MTSEA-biotin (Biotium, 1 mg/ml; 2 μl/μg RNA) in biotinylation buffer (100 mM Tris, 10 mM EDTA pH 7.4, 1 μl was used per μg RNA) for 45 min at RT. An equal volume of chloroform was added, mixed, and incubated with biotinylated RNA for 3 min. The mixture was separated in prespun Phase Trap Gel heavy tubes (5PRIME) (5 min, 16000 rpm). For RNA precipitation and removal of unincorporated MTSEA-biotin, a 1/10 volume 5 M NaCl and an equal volume of isopropanol were added to the aqueous phase and centrifuged (20 min, 16 000 rpm, 4°C). The pellet was washed in an equal volume of 75% ethanol and centrifuged (5 min, 8000 rpm, 4°C) and resuspended in 100 μl ddH_2_O. For separation, untagged and 4sU-tagged RNA was heated (10 min, 65°C) and cooled on ice (5 min). RNA was incubated with 50 μl streptavidin-coated magnetic beads (Miltenyi) (RT, 15 min with rotation). The reaction was applied to μMACs columns (Miltenyi), placed on an μMACS Seperator magnetic stand (Miltenyi), and equilibrated with 900 μl μMACS washing buffer (100 mM Tris, 10 mM EDTA, 1 M NaCl, 0.1% Tween-20, pH 7.5). The columns were washed with 800 μl μMACS washing buffer 5 times. 4sU-biotin-streptavidin-tagged RNA was eluted with 200 μl DTT (100 mM) into 100 μl ddH_2_O. 4sU-tagged RNA was recovered using TRIzol. 4sU-tagged RNA was separated on a 1.5% agarose-gel, containing 5% formaldehyde. RNA quality was assessed under UV-light. 50 ng 4sU-tagged RNA was subjected to cDNA synthesis using SuperScript III Reverse Transcriptase and region-specific primers (Supplementary Table S1).

### J2 RNA immunoprecipitation (J2 RIP)

Cells were harvested, washed in cold PBS and incubated in 5 volumes lysis buffer (100 mM KCl, 5 mM MgCl_2_, 10 mM HEPES pH 7.0, 0.5% NP-40, 1 mM DTT, 100 U RNase inhibitor (Ribolock, Thermo), 1× protease/phosphatase inhibitor cocktails, Roche) for 10 min on ice in presence of DNase I (10U/μl, Sigma) and centrifuged (10 min, 13 000 rpm). Total RNA from 10% of lysate (input) was resuspended in TRIzol. Remaining supernatant was incubated with 5 μg dsRNA antibody J2 (Scicons) at 4°C overnight. Immune complexes were pulled down at 4°C with 40 μl of protein A/G agarose beads (Merck-Milipore) for 45 min at 4°C and washed 4 times in lysis buffer. Total and immunoselected RNA was purified using TRIzol. For detection of immunoselected RNA by autoradiography, RNA was end-labeled, re-extracted, size-separated, transferred and detected as described above.

For qualitative analysis, samples were mixed with 1 volume 2× urea dye (7 M urea, 0.05% xylene cyanol, 0.05% bromophenol blue), incubated at 75°C for 10 min and separated for 30 min at 350 V in 1× TBE buffer (90 mM Tris, 90 mM boric acid, 2 mM EDTA) on a 6% PAGE gel with 7M urea. Gels were stained in 1× TBE buffer containing 1× SYBR gold nuclei acid gel staining solution (Thermo) for 20 min protected from light. RNA was visualized under UV light using a transilluminator (UVP). For quantitative analysis, immunoselected RNA was reverse-transcribed with SuperScript III Reverse Transcriptase using region-specific primers. cDNA was quantified by comparative analysis using real-time PCR. J2 RIP specificity was tested by digestion with recombinant RNaseIII (NEB, 2U) (20 min, RT) in presence of 1× RNase reaction buffer (NEB) prior to immunoprecipitation.

### Northern blot hybridization

RNA associated with MNase-digested, solubilized chromatin was isolated from cells using TRIzol, recovered in 20 μl urea dye, incubated (10 min, 75°C) and separated on a 6% PAGE gel with 7M urea. Separated RNA was transferred on a positively charged nylon membrane (Hybond N+, GE Healthcare) at 300 mA for 30 min using 1× TBE buffer (90 mM Tris, 90 mM boric acid, 2 mM EDTA) and a semi-dry transfer chamber (Invitrogen), washed in ddH_2_O and UV-crosslinked using Stratalinker 2400 (Stratagene) optimal crosslink setting (120 000 mJ/cm^2^). Crosslinked RNA was prehybridized in ULTRAhyb Ultrasensitive hybridization buffer (Invitrogen) (4 h, 42°C) in a hybridization oven (Thermo). Migration fronts of xylene cyanol and bromophenol blue were marked and used as size markers.

For detection of crosslinked RNA, a set of DNA oligonucleotide probes (2 μl of equimolar probe mixture, 10 μM each, Supplementary Table S2), complementary to the *Asi*SI-ER cleavage site DS1, was incubated with 10 μl T4 PNK-containing labeling mix (20 min, 37°C with rotation). End-labeled DS1 probe mix was diluted in 40 μl TE buffer (10 mM Tris, 1 mM EDTA), purified by centrifugation (5 in, 3200 rpm) using preequilibrated Microspin G-25 columns (Invitrogen), boiled (95°C, 5 min), added to hybridization tubes, containing prehybridized membranes, and incubated (36 h, 42°C). Membranes were washed 2× in 1× SSC buffer (15 mM sodium citrate, 150 mM NaCl) containing 0.1% SDS for 10 min at 42°C with rotation, air-dried and subjected to autoradiography. Signals were quantified using ImageJ (NIH).

For loading control, 28S and 18S ribosomal RNA containing total RNA was purified from comparable amounts of cells using TRIzol, resuspended in 2x RNA loading dye (50% formamide, 15% formaldehyde, 1× MOPS buffer, 0.1% bromophenol blue, 10 μg/ml ethidium bromide), incubated (10 min, 75°C) and separated on a 1% agarose gel, containing 6% formaldehyde and 1× MOPS buffer (10 min, 100V), and visualized by ethidium bromide staining under UV-light.

### Single-molecule RNA fluorescence in situ hybridization (sm RNA FISH)

Cells were fixed with 3% formaldehyde in PBS for 20 min, washed 2× in PBS and permeabilized in 70% ethanol overnight at 4°C. Cells were washed in PBS and incubated in 1× SSC buffer containing 15% formamide (15 min, RT). Cover slips were laid on top of 100 μl hybridization mix (5 μl 20× SSC buffer, 1.7 μl yeast transfer RNA (20 mg/ml), 15 μl 100% formamide, 2 μl sm RNA FISH duplexes (20 ug/ml), 1 μl BSA (20 mg/ml), 1 μl RNase inhibitor (Thermo), 26.5 μl 40% dextran sulphate, 47.8 μl ddH_2_O) and incubated (42°C, overnight) in a light-protected humidified chamber. Cover slips were washed in 1× SSC buffer with 15% formamide for 30 min, rinsed in PBS and mounted in 10 μl Mowiol prior to image acquisition by confocal microscopy, using Z-stack sectioning and normalization software for identification of the signal over the average number of pixels in each section. More than 50 cells were analysed for each condition.

For generation of single-molecule RNA FISH duplexes, a set of primary DNA oligonucleotide probes (2 μl equimolar mixture, 10 μM each, Supplementary Table S2) containing a target-specific region and a common linker sequence were hybridized with 1 μl Alexa Flour 488-conjugated secondary probe (100 μM), complementary to the linker sequence, 1 μl 10× NEB buffer 3 and 6 μl ddH_2_O in a PCR machine (1 cycle: 95°C, 3 min; 62°C, 5 min; 25°C, 5 min). Duplexes were kept on ice and protected from light prior to addition to the hybridization mixture.

### RNase *in vitro* digestion

Digestion with structure-specific RNases was performed as described (28). Cells were permeabilized with PBS/0.3% Tween-20 (10 min, RT), washed 1× in PBS and incubated for 20 min at RT with either BSA (Sigma, 0.2 μg/ml final conc., diluted in PBS containing 0.02 mM NaOAc and 0.2 mM Tris), RNaseA (Sigma, 0.2 μg/ml final conc., diluted in PBS containing 0.02 mM NaOAc and 0.2 mM Tris) or RNaseIII (NEB, 2U final conc., diluted in RNase-free H_2_O containing 1× commercial reaction buffer (NEB) prior to fixation. Cells were washed 2× in cold PBS containing RiboLock RNase inhibitor (Thermo, 100 U final conc.), fixed in 3% formaldehyde (8 min, RT) and stained.

For complementation, permeabilized and RNaseA-digested cells were pre-incubated with PBS containing RiboLock RNase inhibitor (Thermo, 100U final conc.) and α-AM (2 μg/ml final conc.) (10 min, RT). Cells were then incubated for additional 20 min at RT with PBS containing RiboLock RNase inhibitor (Thermo, 100 U final conc.) and α-AM (2 μg/ml final conc.) and 50 μg total RNA or 50 μg total RNA, which was immuno-depleted with 5 μg antibodies that recognize dsRNA, DNA–RNA hybrids or ssDNA prior to incubation. Total or immuno-depleted RNA was purified using acidic phenol/chloroform extraction. Cells were washed 1× in cold PBS containing RiboLock RNase inhibitor (Thermo, 100 U final conc.), fixed in 3% formaldehyde (8 min, RT) and stained.

### Quantitation of DNA double-strand breaks

Induction of DSBs was quantified as described (36). Genomic DNA from comparable amounts of cells cultured in absence or presence of 4OHT, or preincubated with α-AM (2 μg/ml) for 20 h before addition of 4OHT, was purified and on-column digested with RNaseA using Wizard SV genomic DNA purification kit (Promega). Levels of non-restricted genomic DNA were measured as Ct-values by quantitative PCR (qPCR) using region-specific primers (Supplementary Table S1), which either amplify genomic DNA across the two *Asi*SI-ER sites acDS-I (spanning promoter-associated *Asi*SI-ER site DS1) and acDS-II (spanning a genic *Asi*SI-ER site in the *CYB561D1* gene) or amplify one non-restricted control locus (noDSB) or two non-restricted housekeeping genes (*ACTB, GAPDH*). Ct-values were transformed into relative fold-change using the ΔΔcT method. Therefore, Ct-values measured across *Asi*SI-ER sites or at the non-restricted control locus in absence or presence of drugs were first normalized to Ct-values measured at either of the two housekeeping genes in absence or presence of drugs. The normalized Ct-values measured in presence of 4OHT or after preincubation with α-AM were again normalized to Ct-values measured in absence of 4OHT for the two cut *Asi*SI-ER sites and the non-restricted control locus. Double-normalized cT values were calculated as fold-changes by logarithmic transformation with values in absence of 4OHT set to 1. To plot % of DSBs, values in absence of 4OHT, i.e. ‘1’ were set to ‘0’ and values in presence of drugs were transformed into % of lost signal by calculating (1-value)*100%.

### mNET-sequencing

Three biological replicates were pooled and submitted to small RNA-seq library preparation. Library preparation was performed using the TruSeq small RNA preparation kit (Illumina) as described (35). mNET-seq data were processed as described (37). Briefly, mNET-seq adapters were trimmed with Cutadapt v. 1.8.3, (https://cutadapt. readthedocs.io/en/stable/) in paired end mode and mapped to the human hg38 reference genome with Tophat v. 2.0.13, (https://ccb.jhu.edu/software/tophat/), with the use of the following parameters: -g 1 -r 3000 –no-coverage-search. Only properly paired and mapped reads were used for downstream analysis (samflags 0×63, 0×93, 0×53, 0xA3, extracted with SAMtools v. 1.2, http://www.htslib.org/). Single nucleotide profiles were generated by extracting most 3′ nucleotide of the second read and the strandedness of the first read with a custom perl script. Trackhubs in the UCSC browser were created by employing the UCSC bedGraphToBigWig tool. *Asi*SI-ER sites were mapped to the hg38 genome in R with the matchPattern function from the Biostrings package. 94 efficiently restricted genic *Asi*SI sites (as above), were used for metagene profiles. To compare, γH2A.X /H2A.X–log2-signal (38) was computed with deepTools2 bamCompare. 94 sites in genes with the lowest γH2A.X /H2A.X-log_2_-signal were extracted to serve as negative (uncut) control metagene profiles. The average coverage across the 94 sites in each position is depicted. 0 refers to the 5′end of the *Asi*SI-ER site. A rolling average of 5nt was taken to smooth the data slightly. The rolling of 5 nt means that the average signal over five positions is taken (for the nucleotide x this means the average of the window [x,x+4]). Graphs were generated using ggplot2 (http://www.ggplot2.org/) in R (http://www.R-project.org).

## RESULTS

### Promoter-associated DSBs engage CTD Tyr1-phosphorylated RNAPII

**Figure 1.**
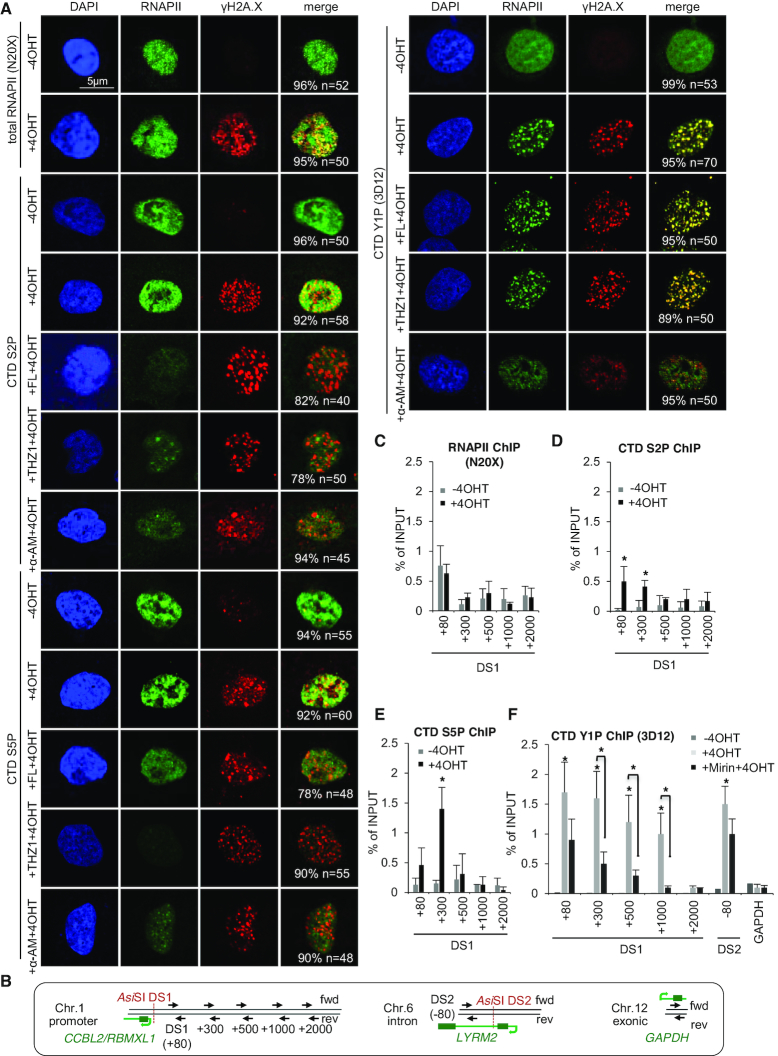
Elevated RNAPII CTD Tyr1 phosphorylation at DSBs. (**A**) Imaging of total or phospho-CTD specific RNAPII variants. γH2A.X, S139 H2A.X phosphorylation; 4OHT, 4-Hydroxytamoxifen; FL, Flavopiridol; α-AM, α-Amanitin. n, number of cells with shown phenotype in %. (**B**) Structure of *Asi*SI-ER site DS1/2 (red dashed line), qRT-PCR primer with distance from target site in nts (black arrow, fwd/rev forward/reverse primer); protein-coding gene promoter (green arrow), exon (green box), intron (green line). (**C–F**) ChIP analysis of (C) total (N20X), (D) CTD S2P, (E) CTD S5P, or (F) CTD Y1P RNAPII occupancy at DS1 using site-specific primers. (C–F) Asterisk, *P*-value < 0.05, two-tailed *t*-test. Error bar: mean ± SEM, *n* = 3.

To study RNAPII in response to DSBs, we employed the *Asi*SI-ER endonuclease, which is constitutively expressed, fused to the estrogen receptor ligand binding domain and activated by 4-hydroxytamoxifen (4OHT) in human U2OS cells (19). The human genome contains 1231 predicted, locus-specific *Asi*SI-ER cleavage sites (Supplementary Figure S1A). 859 sites are located in genic regions. By assessing histone H2A.X phosphorylation (γH2A.X), previous work showed that ∼100 loci are efficiently cut by *Asi*SI-ER *in vivo* (39). We observed a time-dependent increase in γH2A.X levels, but no significant change in total RNAPII levels or CTD phospho-marks in response to 4OHT (Supplementary Figure S1B). However, a subset of RNAPII molecules, particularly phosphorylated at CTD Tyr1 residues (CTD Y1P) was enriched at γH2A.X foci (Figure 1A and Supplementary Figure S1C). CTD S2/5P staining was sensitive to preincubation with Flavopiridol or THZ1, which inhibit CTD phosphorylating cyclin-dependent kinase 9 (Cdk9) and Cdk7, respectively. Preincubation with α-Amanitin (α-AM), which directly inhibits RNAPII and triggers its degradation, diminished all CTD marks at DSBs. We confirmed suppression of CTD S2/5P, but not Y1P or total RNAPII by Flavopiridol as well as depletion of RNAPII by α-AM and induction of γH2A.X levels by 4OHT in presence of RNAPII inhibitors on immunoblots. Inhibition of CDK7, which indirectly regulates S2P levels by activating CDK9 (40), globally reduced CTD S2/5P hyper-phosphorylated RNAPII levels (Supplementary Figure S1D). Furthermore, we confirmed enrichment of CTD Y1P at γH2A.X and 53BP1 foci in various cell lines (Supplementary Figure S1E). To assess RNAPII occupancy at DSBs, we employed ChIP analysis upstream of a previously assessed *CCBL2/RBMXL1* promoter-associated *Asi*SI-ER site (DS1) (39), an intronic site within the *LYRM2* gene (DS2) (41) and a non-restricted exonic site (*GAPDH*) (Figure 1B). We detected no significant increase in total RNAPII occupancy up to 2kb upstream of DS1 in presence of 4OHT (Figure 1C), suggesting that steady-state, promoter-associated RNAPII levels at DS1 are not affected by *Asi*SI-ER cleavage. However, analysis of CTD-phosphorylated RNAPII subpopulations revealed a 3-5-fold increase in CTD S2/5P signals up to 300nt upstream of DS1 (Figures 1D and 1E). Strikingly, CTD Y1P occupancy increased >5-fold up to 1 kb distant from DS1, at DS2, but not at the *GAPDH* locus (Figure 1F). We conclude that phosphorylated RNAPII, in particular Y1P, associates with DSBs.

Early events in DSB repair involve recognition of DNA ends by the Mre11-Nbs1-Rad50 (MRN) complex (42), which may recruit RNAPII to DSBs (27). Thus, we assessed whether formation of CTD Y1P foci requires MRN. We detected strong colocalization of CTD Y1P with Mre11 and 53BP1, which was sensitive to depletion of Mre11 (Supplementary Figures S2A and B). Mre11 depletion had no significant impact on total RNAPII or CTD phospho-marks, but prevented formation of Mre11 or γH2A.X foci (Supplementary Figures S2C and D). Next, we assessed CTD Y1P chromatin occupancy after preincubation with Mirin, an inhibitor of Mre11 exonuclease activity. Mirin treatment significantly reduced CTD Y1P occupancy at DS1/2 (Figure 1F), with little impact on total RNAPII or CTD Y1P levels (Supplementary Figure S2E). Mirin also increased the number of cells in G_2_-phase (Supplementary Figure S2F), as described (43).

As DSBs may trigger *de novo* RNAPII activity to stimulate foci formation (27), we assessed the formation and number of 53BP1 and MDC1 foci in presence of RNAPII inhibitors (Supplementary Figures S3A–D). Preincubation with Flavopiridol neither impaired recruitment of DDR factors, nor formation of high numbers of foci (*n* > 10) in 70–90% of cells. α-AM, instead, impaired formation of MDC1 foci in >80% of damaged cells (Supplementary Figure S3E). Importantly, RNAPII inhibitors did not induce DSB foci *per se*. To control for induction of DSBs in presence of α-AM, we confirmed that cleavage of DS1 and another genic *Asi*SI-ER site in the *CYB561D1* gene was not altered by α-AM (Supplementary Figure S3F). Further, α-AM did not alter expression of MDC1, 53BP1, the proliferation marker Ki-67 or *Asi*SI-ER itself (Supplementary Figure S3G) or cell cycle distribution (Supplementary Figure S3H). We conclude that damage-induced CTD Y1P foci depend on the MRN complex.

### c-Abl catalyses formation of CTD Y1P foci at promoter-associated DSBs

**Figure 2.**
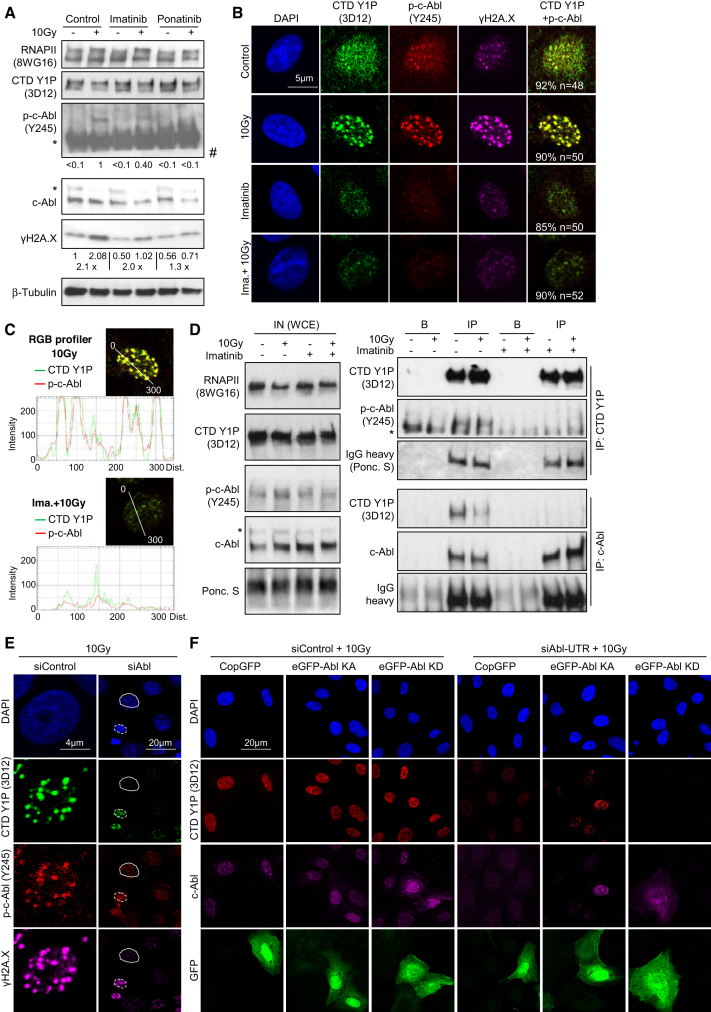
Requirement of c-Abl for CTD Y1 foci formation at DSBs. (**A**) Immunoblots detecting total RNAPII, CTD Y1P, total c-Abl, phospho-c-Abl (p-c-Abl, Y245) and γH2A.X. β-Tubulin, control; 10 Gray (Gy), dose of irradiation; asterisk, unspecific; #, cut. (**B, C**) Imaging and RGB quantitation of CTD Y1P and p-c-Abl. *n*, number of cells with shown phenotype in %. (**D**) Immunoblots detecting CTD Y1P, p-c-Abl and total c-Abl in input (IN) or upon immunoprecipitation with antibodies (IP) or beads only (B). Controls: total RNAPII (8WG16), Ponceau S, immunoglobulin chains (IgGs); asterisk, unspecific. (**E**) Imaging of CTD Y1P, p-c-Abl and γH2A.X upon transfection with siRNA specific for c-Abl. Scrambled siRNA, control. Representative images are shown. Full/broken circles, c-Abl-depleted/non-depleted nuclei. (**F**) as in (E), upon cotransfection of untranslated region-specific c-Abl siRNA (siAbl-UTR) with enhanced GFP-tagged c-Abl kinase active/kinase dead constructs (eGFP-Abl KA/KD) or copepod GFP expressing vector (CopGFP). Scrambled siRNA, control. Representative images are shown.

Besides MRN, the Phosphatidylinositol 3-kinase-related kinases (PIKKs) *Ataxia Telangiectasia mutated* (ATM), ATM-related (ATR) and DNA-dependent protein kinase (DNA-PK) govern the DDR by targeting hundreds of substrates (44–47). We speculated that CTD Y1P foci depend on PIKKs. Indeed, inhibition or knockdown of PIKKs not only impaired the formation of γH2A.X and 53BP1 foci, but also strongly reduced CTD Y1P foci, but not CTD Y1P levels (Supplementary Figures S4A-E). Interestingly, ionizing radiation activates the tyrosine kinase c-Abl in a DNA-PK- and ATM-dependent manner (48–51). A dose of 10 Gray induced phosphorylation of c-Abl and γH2A.X, but not CTD Y1P in absence of c-Abl inhibitors Imatinib or Ponatinib (Figure 2A). c-Abl colocalized with CTD Y1P and γH2A.X foci in irradiated nuclei, which was partially impaired by Imatinib (Figures 2B and C, Supplementary Figures S5A and B). Next, we applied irradiation kinetics. Again, we detected a time- and dose-dependent induction of γH2A.X, but not CTD Y1P levels (Supplementary Figures S5C and D) and a dose-responsive formation of CTD Y1P and γH2A.X foci, which partially colocalized with p-c-Abl (Supplementary Figure S5E). Moreover, p-c-Abl co-immunoprecipitated with CTD Y1P in an Imatinib-sensitive manner (Figure 2D and Supplementary Figure S5F). Next, we applied RNAi to knockdown c-Abl. Similar to Imatinib, depletion of c-Abl did not affect CTD Y1P levels (Supplementary Figure S6A), but prevented formation of CTD Y1P and γH2A.X foci in irradiated nuclei (Figure 2E and Supplementary Figure S6B) or upon induction of *Asi*SI-ER (Supplementary Figure S6C). To perform complementation experiments, we utilized siRNA resistant, GFP-tagged c-Abl kinase active or kinase dead (eGFP-Abl KA or KD) constructs and a UTR-specific c-Abl siRNA in a knockdown-knockin approach. When re-expressing eGFP-Abl or GFP control in absence or presence of endogenous c-Abl, we found that eGFP-Abl constructs did not alter CTD Y1P levels (Supplementary Figure S6D). Strikingly, however, presence of eGFP-Abl KA, but not eGFP-Abl KD rescued formation of CTD Y1P foci in c-Abl-depleted, irradiated nuclei (Figure 2F and Supplementary Figure S6E). Collectively, we conclude that c-Abl directly associates with RNAPII to promote formation of CTD Y1P foci at DSBs.

### CTD Y1P produces strand-specific, damage-responsive transcripts

**Figure 3.**
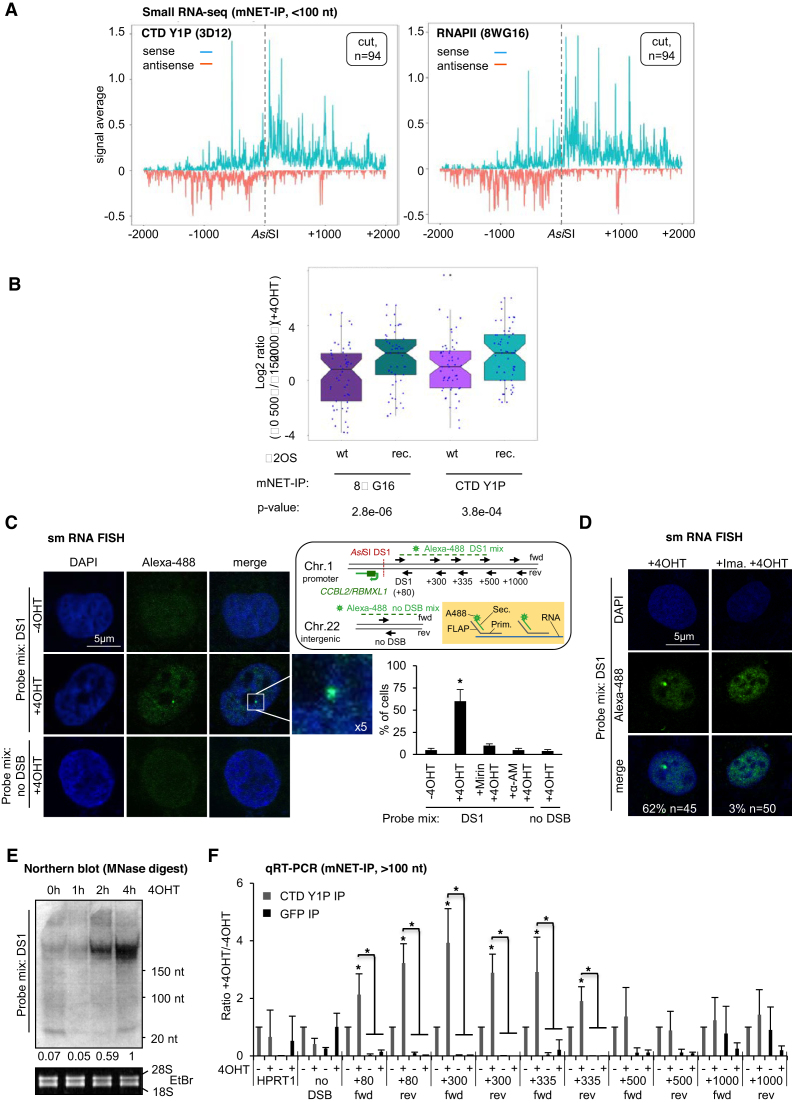
DART production at DSBs. (**A**) mNET-seq depicting average sense/antisense signals across 94 genic cut *Asi*SI-ER sites. Left/right, CTD Y1P/total RNAPII (8WG16) IP; dashed line, *Asi*SI site. (**B**) Box plot showing mNET-seq signals up to 500 nts distant from 94 cut *Asi*SI-ER sites normalized to downstream signals (1500–2000 nts) for wild type U2OS (wt) and *Asi*SI-ER U2OS (rec.) cells. Horizontal black line, median. (**C**) Imaging of RNA originating up to 500 nts distant from DS1 using locus specific probe mix DS1. White box, 5× zoom (left). Probes were coupled with a secondary, Alexa-488 (A488)-conjugated probe by hybridization to a unique, non-hybridizing overhang (FLAP). See scheme for details (top right). Quantitation depicts number of A488-positive nuclei in % (bottom right), *n* > 50. Asterisk, *P*-value < 0.05, two-tailed *t*-test. Error bar: mean ± SEM, *n* = 3. (**D**) as in (C) upon incubation with Imatinib. *n*, number of cells with shown phenotype in %. (**E**) Northern blot of chromatin-associated RNA using end-labeled probe mix DS1 for hybridization. Ethidium bromide (EtBr) staining of 28S/18S rRNA, control. (**F**) qRT-PCR of reverse-transcribed RNA, associated with CTD Y1P at DS1 using site-specific primers. Asterisk, *P*-value < 0.05, two-tailed *t*-test. Error bar: mean ± SEM, *n* = 3.

To assess CTD Y1P activity at DSBs, we analysed RNA levels at DS1. Using total RNA, we measured a minor, 4OHT-induced and Mirin-sensitive increase in forward-oriented transcripts, generated by RNAPII activity upstream of, and toward the *CCBL2/RBMXL1* promoter, but not at uncut exonic (*HPRT1*) or intergenic (no DSB) loci (Supplementary Figure S7A). To assess nascent transcripts, we applied 4-thiouridine-(4sU) labeling. Again, we measured a 2-fold increase in forward-oriented RNA in presence of 4OHT (Supplementary Figure S7B). 4sU-tagged ribosomal RNA was reduced by 4OHT incubation (Supplementary Figure S7C). However, 4OHT did not alter reverse-oriented transcript levels, arguably reflecting cryptic-unstable/promoter-upstream transcripts (52). Next, we immunoprecipitated mammalian nascent elongating transcripts (mNETs), which are directly associated with, and protected by transcribing RNAPII (35), using phospho-CTD selective antibodies. We detected constant association of mNETs with CTD Y1P by autoradiography in 4OHT induction kinetics (Supplementary Figure S7D). We separated CTD Y1P-associated RNA into small (<100nt) and long (>100nt) fractions (Supplementary Figures S7E and F) and performed small RNA-seq. Strikingly, we observed robust mNET-seq traces, reflecting CTD Y1P activity, on both sense and antisense strands up to 1000bp distant from cut, but not uncut genic *Asi*SI-ER sites, which was confirmed by using the total RNAPII antibody 8WG16 (Figure 3A and Supplementary Figure S7G). We also compared mNET-seq signals for 94 cut and uncut sites in *Asi*SI-ER expressing with the same 94 sites in wild type U2OS cells (Supplementary Figures S7H, S8 and S9; GEO accession number GSE96825). Again, we detected prominent RNAPII activity around cut genic, but not intergenic *Asi*SI-ER sites. We also detected mNET-seq traces in wild type cells, confirming that the majority of 94 genic *Asi*SI-ER sites are associated with active promoters. Importantly, however, mNET-seq signals in *Asi*SI-ER expressing cells were significantly elevated compared to wild type (Figure 3B). To visualize 4OHT-induced nascent RNA, we employed RNA FISH by targeting forward-oriented nascent RNA that originates upstream of the *CCBL2/RBMXL1* promoter (Figure 3C and Supplementary Figure S10A). We detected nuclear foci in ∼50% of cells, which were sensitive to Mirin and α-AM. Importantly, no foci were detected at a non-restricted control locus, suggesting RNA-specific detection with probes. Imatinib also impaired the 4OHT-induced detection of RNA at DS1 (Figure 3D and Supplementary Figure S10B). We also observed a time-dependent accumulation of forward-oriented chromatin-associated RNA at DS1 by northern blotting (Figure 3E). When applying qRT-PCR on the long fraction of CTD Y1P-associated RNA, we detected a 2-4-fold induction of forward- and reverse-oriented transcripts up to 300 nts distant from DS1 (Figure 3F). We conclude that CTD Y1P generates DSB-induced RNA, which we term primary (away from DSBs) and secondary (toward DSBs) damage-responsive transcripts (pri-/se-DARTs).

### DNA–RNA hybrids link DARTs synthesis to formation of dsRNA, recruitment of 53BP1 and MDC1 to DSBs and efficient DSB signalling

**Figure 4.**
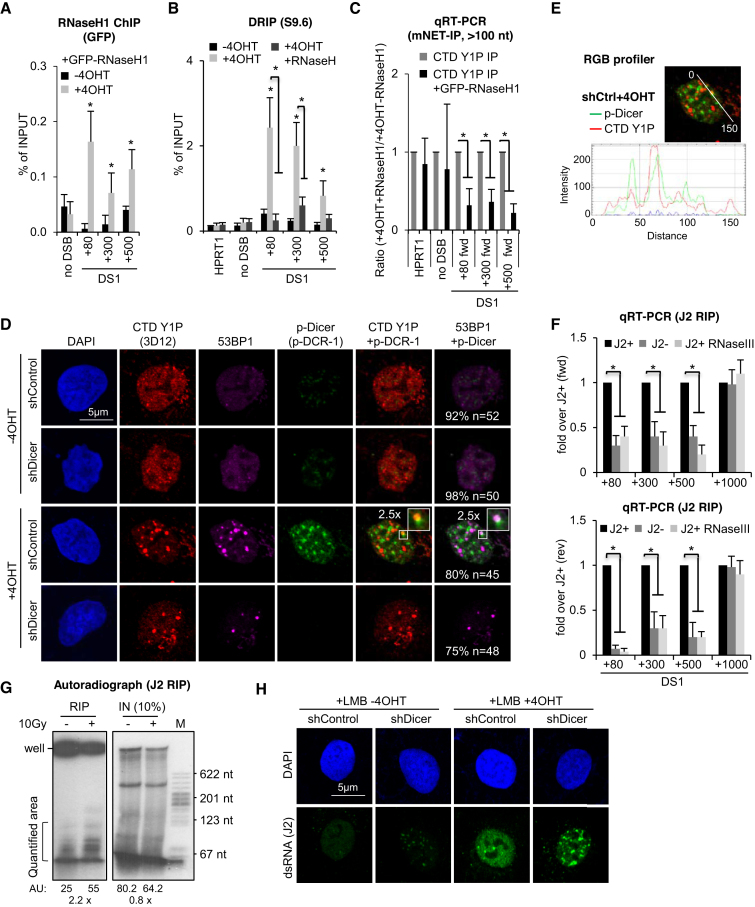
Formation and p-Dicer-dependent turnover of dsRNA at DSBs. (**A**) ChIP analysis of GFP-RNaseH1 occupancy at DS1 using site-specific primers. (**B**) Quantitative real-time PCR (qRT-PCT) of DNA immunopurified from DNA–RNA hybrids (DRIP) or upon incubation with recombinant RNaseH at DS1 using S9.6 hybridoma supernatant and region-specific primers. (**C**) qRT-PCR analysis of transcripts associated with CTD Y1P and immunopurification (mNET-IP). Values were normalized to data in absence of GFP-RNaseH1. (A–E) Asterisk, *P*-value < 0.05, two-tailed *t*-test. Error bar: mean ± SEM, *n* = 3. (**D, E**) Imaging and RGB quantitation of CTD Y1P and p-Dicer (p-DCR-1). White box, 2.5× zoom; *n*, number of cells with shown phenotype in %. (**F**) qRT-PCR of cDNA after J2 immunoselection with (J2 RIP) and reverse transcription with forward (upper panel)- and reverse (lower panel)-oriented primers spanning a region up to 1000 nts distant from DS1 in presence (J2+) or absence (J2-) of 4OHT, or upon preincubation of lysed material with recombinant RNaseIII (J2+ RNaseIII) prior to J2 RIP. J2+ values were set to 1. Asterisk, p-value <0.05, two-tailed t-test. Error bar: mean ±SEM, *n* = 3. (**G**) Autoradiograph detecting J2 immuno-selected (RNA IP) or total (IN) RNA or pBR322 MspI digest (M) upon end-labeling and PAGE separation. AU, arbitrary units. (**H**) as in (D), but preincubated with Leptomycin B (LMB) and stained with J2. Representative images are shown.

**Figure 5.**
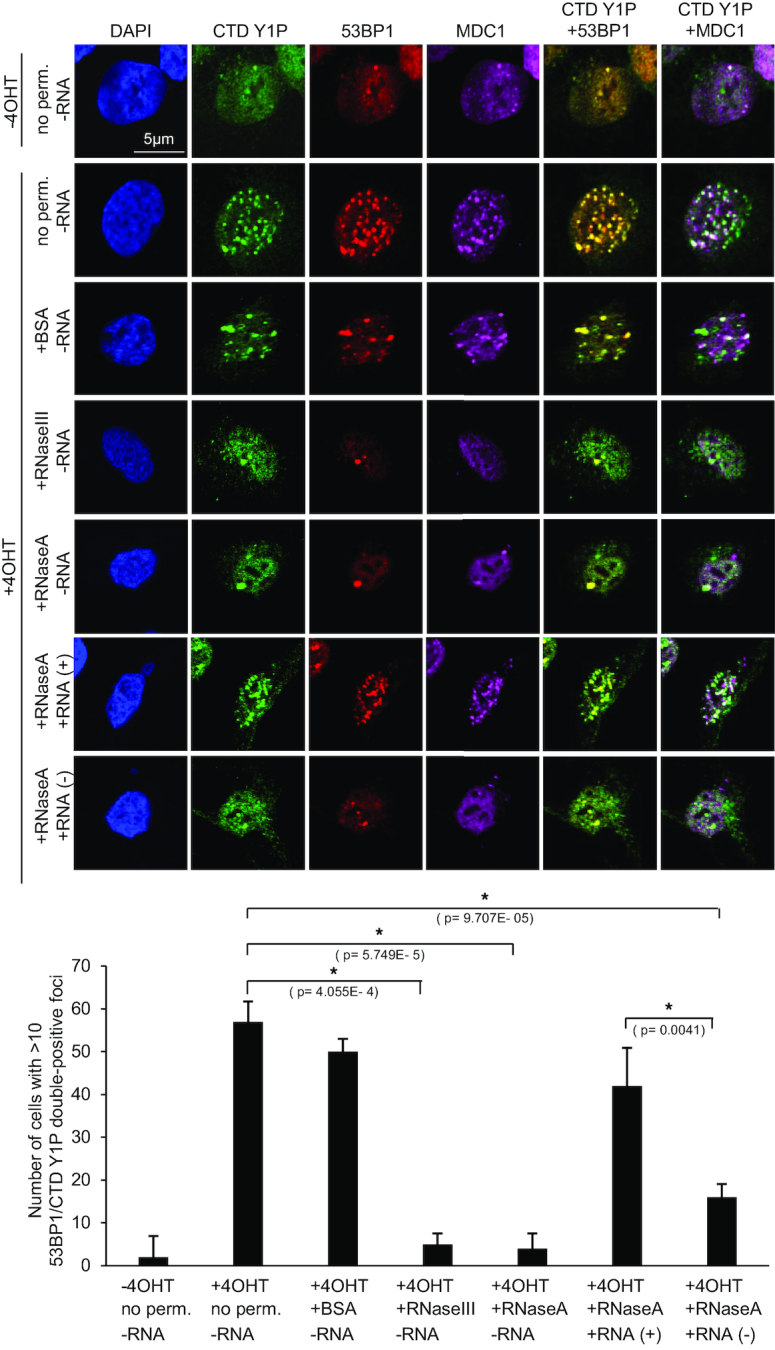
dsRNA-dependent recruitment of 53BP1 and MDC1 to DSBs. Imaging of CTD Y1P, 53BP1 and MDC1 without permeabilization, upon permeabilization and incubation with bovine serum albumin (BSA, control) or structure-specific RNases prior to fixation, or upon add-back with total RNA purified from *Asi*SI-ER U2OS cells in presence (+) or absence (–) of 4OHT. Representative images are shown (top). Quantification of cells with foci staining double-positive for CTD Y1P and 53BP1 (bottom). The experiment was done in triplicates. 60 cells were counted per replicate. Cells were scored based on average number *n* < 10 of CTD Y1P/53BP1 double-positive foci (yellow) per cell. Asterisk, *P*-value < 0.05, one-tailed *t*-test. Error bar: mean ± SEM, *n* = 3.

**Figure 6.**
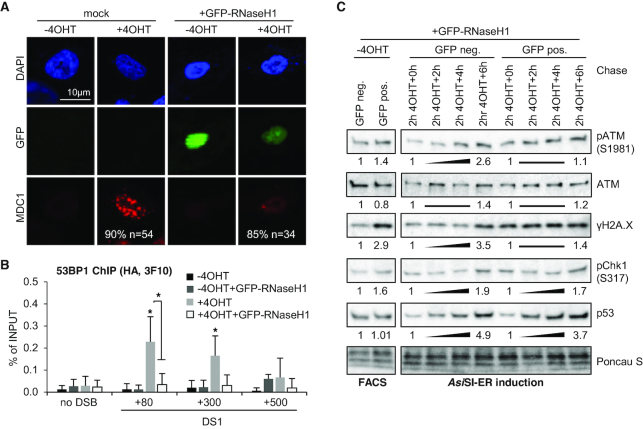
DNA–RNA hybrid-dependent DSB foci formation and onset of DSB signalling. (**A**) Confocal imaging of MDC1 and GFP-RNaseH1 following transient transfection of pEGFP-M27 or non-transfected (mock) cells. *n*, number of cells with shown phenotype in %. (**B**) ChIP analysis of HA-53BP1 occupancy at DS1 using HA antibody (3F10) and site-specific primers. Asterisk, *P*-value < 0.05, two-tailed *t*-test. Error bar: mean ± SEM, *n* = 3. (**C**) Immunoblots detecting phospho-ATM, total ATM, γH2A.X, phospho-Chk1 and p53 levels following fluorescence-activated cell sorting (FACS) in absence of 4OHT (left) and after pulse-chase 4OHT incubation kinetics (right). Ponceau S, control.

Recent evidence suggests that DNA–RNA hybrids stimulates DSB repair (53). Transient, strand-specific DNA–RNA hybrids form at a subset of DSBs to promote transcription-associated homologous recombination (HR) in *S. pombe* (54) and humans via recruitment of Rad52, BRCA1 (55) and MDC1 (56,57). To test for formation of DNA–RNA hybrids at DS1, we expressed GFP-tagged RNaseH1, which processes RNA when hybridized to DNA, in *Asi*SI-ER U2OS cells and employed ChIP analysis to assess GFP-RNaseH1 occupancy in response to 4OHT incubation. We measured a 2-3-fold increase in GFP occupancy at DS1 in presence of 4OHT (Figure 4A). We also employed the hybrid-specific S9.6 antibody in DNA–RNA immunoprecipitation (DRIP). In line with published DRIP-seq data (56), we detected a 2-3-fold increase in signals next to DS1 in presence of 4OHT, which were sensitive to *in vitro* RNaseH digestion (Figure 4B). However, formation of DNA–RNA hybrids at DSBs remains largely enigmatic. We previously showed that *Asi*SI-ER cleavage triggers accumulation of dsRNA, which is processed by nuclear, DSB-associated p-Dicer to promote 53BP1 and MDC1 recruitment (29). Given that Dicer utilizes a variety of nuclear dsRNA substrates (58) and that formation of DNA–RNA hybrids favours accumulation of Dicer-dependent dsRNA at some mammalian terminators (33), we hypothesized that DNA–RNA hybrids may stimulate initiation of CTD Y1P-dependent reverse complementary RNA (seDARTs) to form dsRNA. To assess the impact of DNA–RNA hybrids on CTD Y1P-associated transcripts, we compared levels of forward-oriented transcripts, i.e. se-DARTs upon 4OHT incubation at DS1 in absence or presence of GFP-RNaseH1 and found that overexpression of GFP-RNaseH1 partially impaired accumulation of se-DARTs (Figure 4C). Using S9.6 and the p-Dicer-specific antibody p-DCR-1 (29,30), we detected 4OHT-induced, Imatinib-sensitive colocalization of DNA–RNA hybrids with CTD Y1P and MDC1 foci (Supplementary Figure S11A) as well as colocalization of p-Dicer with CTD Y1P and 53BP1 (Figures 4D and E). Dicer depletion modulated γH2A.X levels in absence and presence of DSBs (Supplementary Figure S11B), as described (29). To test for dsRNA formation locally, we employed the dsRNA antibody J2 for RNA IP and quantified immunoselected dsRNA levels derived from DS1 in a strand- and sequence-specific manner. We detected increased, RNaseIII-sensitive RNA levels, containing forward- and reverse-oriented transcripts up to 500nts from DS1 (Figure 4F and Supplementary Figure S11C). To test for dsRNA formation globally, we performed autoradiography with J2 immuno-selected end-labeled RNA and detected 2-3-fold elevated levels of small RNA upon irradiation (Figure 4G). Next, we visualized dsRNA by microscopy. J2 signals accumulated in nuclear foci upon 4OHT incubation, inhibition of nuclear export by Leptomycin B and Dicer depletion, but displayed pan-nuclear reactivity in damaged nuclei expressing Dicer (Figure 4H). We and others confirmed J2 specificity toward dsRNA (>40bp) *in vitro* and *in vivo* (32,59,60). To further validate J2 specificity we subjected total or J2 immuno-selected RNA to digestion with dsRNA-specific RNaseIII and gold staining (Supplementary Figure S11D). While little dsRNA was detectable upon J2 immuno-selection in presence of Dicer, we stained dsRNA of various sizes upon Dicer-depletion, which was sensitive to RNaseIII digestion *in vitro*. However, incubation with 4OHT did not alter the levels of immuno-selected dsRNA. To investigate the relevance of DNA–RNA hybrids and dsRNA for 53BP1 and MDC1 recruitment, we digested permeabilized cells with structure-specific RNases (Figure 5). First, we confirmed that 4OHT-induced colocalization of CTD Y1P with 53BP1 and MDC1 foci is preserved upon treatment with detergent. Digestion with RNaseIII and RNaseA largely abolished foci formation. Next, we added total RNA purified from damaged or normal cells to RNaseA-digested cells. Strikingly, we detected reformation of CTD Y1P, 53BP1 and MDC1 foci in presence of RNA extracted from damaged cells. Moreover, RNA immuno-depleted for RNA hybrids, but not single-stranded (ss)DNA or mock-depleted RNA attenuated foci formation (Supplementary Figure S12A). Next, we investigated the impact of GFP-RNaseH1 expression for recruitment of MDC1 to DSBs. GFP-RNaseH1 expression strongly impaired formation of 4OHT-induced MDC1 foci (Figure 6A). To test the relevance of DNA–RNA hybrids for recruitment of 53BP1 to DSBs, we expressed either HA-tagged, full-length 53BP1 (HA-53BP1) alone or in combination with GFP-RNaseH1 in *Asi*SI-ER U2OS cells and performed ChIP at DS1 (Figure 6B). As expected, GFP-RNaseH1 expression strongly impaired HA-53BP1 occupancy in presence of 4OHT. Co-transfection of HA-53BP1- and GFP-RNaseH1-encoding plasmids partially impaired HA-53BP1 expression. However, addition of 4OHT did not alter levels of ectopically expressed proteins (Supplementary Figure S12B). We next wished to test the relevance of DNA–RNA hybrids for DSB-induced signalling by employing 4OHT incubation kinetics. When enriching for GFP-RNaseH1-expressing cells by FACS sorting (Supplementary Figure S12C), we observed an increase in γH2A.X in absence of 4OHT, but not phosphorylation of ATM or checkpoint kinase 1 (Chk1) or p53 induction (Figure 6C, left panel), suggesting that overexpression of GFP-RNaseH1 may have modestly interfered with DNA replication and caused an increase in pan-nuclear γH2A.X levels during S-phase, but not induction of DSBs. Addition of 4OHT to GFP-negative sorted cells caused a 2-5-fold, time-dependent increase in ATM, H2A.X and Chk1 phosphorylation, accompanied with accumulation of p53. In contrast, phosphorylation of ATM and H2A.X, but not induction of p53, was impaired in GFP-positive sorted cells (Figure 6C, right panel and Supplementary Figure S12D–H). We also repeated 4OHT induction in non-sorted cells (Supplementary Figure S12I). Again, expression of GFP-RNaseH1 impaired phosphorylation of ATM, but also affected γH2A.X levels in presence of 4OHT. We conclude that dsRNA is subject to p-Dicer processing and that formation of DNA–RNA hybrids is required for efficient recruitment of 53BP1 and MDC1 to DSBs and onset of DSB signalling.

## DISCUSSION

**Figure 7.**
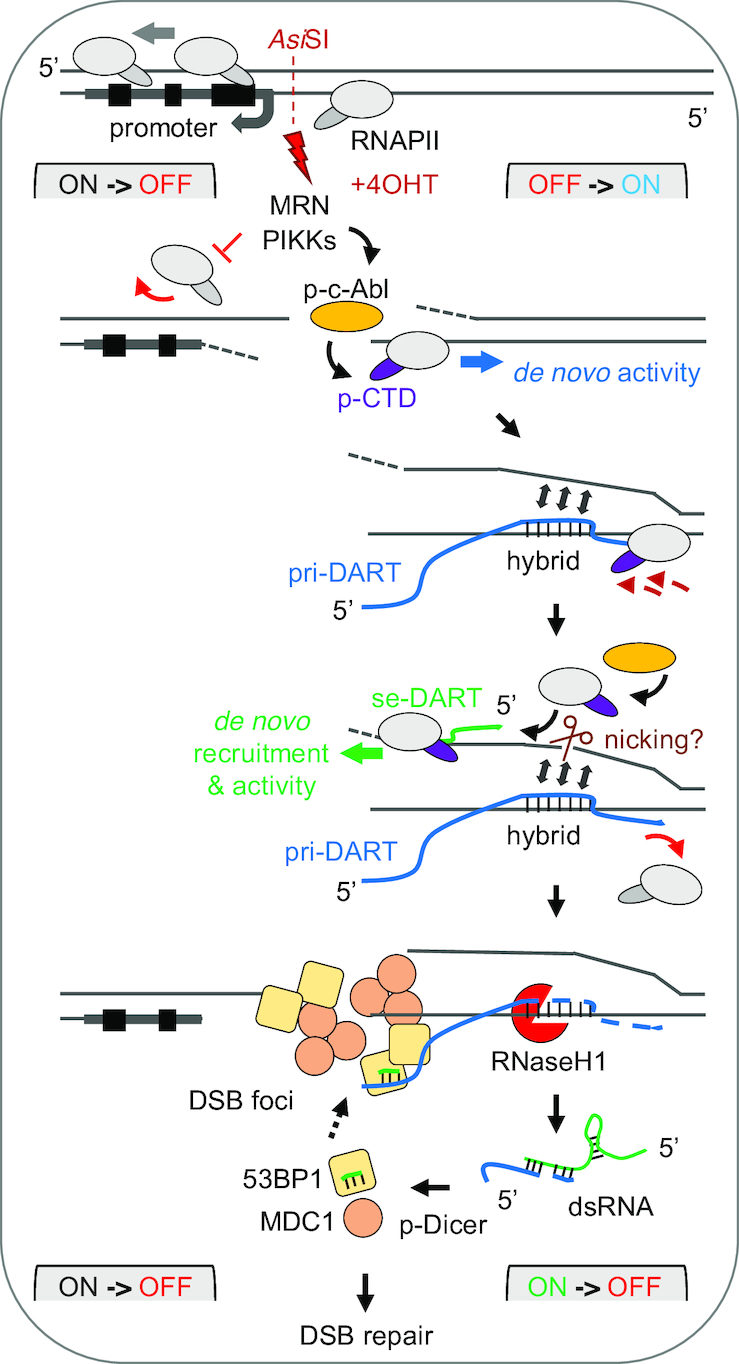
Model for production and processing of damage-responsive transcripts (DARTs) at promoter-associated DSBs. See main text for details.

We provide evidence for CTD Y1P RNAPII activity at promoter-associated DSBs, which results in strand-specific DARTs synthesis and subsequent dsRNA formation (Figure 7). Upon activation of PIKK signalling, c-Abl stimulates the *de novo* activity of promoter-occupying, chromatin-bound RNAPII by placing CTD Y1 phospho-marks. CTD Y1P produces pri-DARTs, which hybridize with template DNA and, in turn, stimulate production of reverse-oriented se-DARTs at minimally resected DNA ends. Upon release from template DNA, se-DARTs form dsRNA with partially processed pri-DARTs. Production of se-DARTs may involve endonucleolytic cleavage of displaced ssDNA and *de novo* recruitment of RNAPII. dsRNA is further processed by p-Dicer to promote recruitment of 53BP1 and MDC1 to lesions and stimulates RNA-dependent DSB signalling. Thus, *Asi*SI-ER cleavage at promoters triggers a switch in transcriptional directionality, which involves transient activation of RNAPII activity prior to gene silencing (61). dsRNA may represent a signal for RNAi-dependent gene silencing in analogy to transcriptional gene silencing in lower eukaryotes (22,62).

How does c-Abl involve RNAPII in DART synthesis? Tyrosine kinases are tightly linked to the DDR (63). c-Abl phosphorylates various HR factors, such as Rad51 (64) and amplifies ATM signalling to stimulate chromatin relaxation via phosphorylation of KAT5/Tip60 acetyltransferase (65). Interestingly, efficient recruitment of Tip60 to DSBs depends on small ncRNA (66). We propose that c-Abl regulates DSB-induced RNA synthesis by catalysing a localized accumulation of CTD Y1P marks. c-Abl is necessary for damage-induced formation of CTD Y1P foci, but may not be the only kinase to place Y1P marks. Both c-Abl and the Abl-related gene product (ARG) can phosphorylate CTD Y1 *in vitro* (51,67). c-Abl is a ubiquitously expressed protein that directly associates with chromatin and DDR factors and its knockout is embryonic lethal (68). ARG, in contrast, is a non-essential, cytoplasmic protein (69), indicating that c-Abl may function as DSB-induced human CTD kinase *in vivo*. Our findings place c-Abl upstream of canonical DSB repair and link it to the recognition of promoter-associated DSBs.

Genetic strategies exist to replace endogenous RNAPII with CTD mutants. Experiments with the human CTD Y/F48 mutant clearly show that Y1P is essential, since RNAPII completely devoid of Y residues is intrinsically instable and causes lethality (5). Intriguingly, the Manley lab recently reported elevated CTD Y1P levels upon various stresses, including DNA damage in *S. cerevisiae*. The budding yeast CTD Y1F mutant is viable and hypersensitive to DNA damage (9). *S. cerevisiae* encodes no typical tyrosine kinases, which causes extremely low steady state tyrosine phosphorylation levels. Thus, stress-induced atypical tyrosine kinases, such as Mpk1/Slt2, may elevate the number of CTD Y1 phospho-marks dramatically, and to an extent quantifiable by 3D12 staining. The complexity of human CTD heptad-repeats together with distinct antibody recognition patterns may impair quantitative detection of human Y1P marks. Indeed, partial mutation of Y1 residues does not seem to alter 3D12 reactivity (70), which is only lost upon complete mutation of Y1 (5,9) or α-AM treatment. Moreover, c-Abl inhibition has little impact on elongating RNAPII (71) and Y1P levels are not completely abolished upon deletion of *MPK1/SLT2* in *S. cerevisiae* (9), pointing toward redundancy in CTD Y1-modifying kinases. We speculate that the bulk of Y1 phosphorylation, mostly present as ‘baseline’ phospho-mark on non-chromatin bound RNAPII, is balanced at steady-state, and placed *de novo* on a certain fraction of chromatin-bound RNAPII in response to DSBs.

Our data suggest a role for the MRN complex and PIKKs in CTD Y1P foci formation and RNA synthesis at DSBs. MRN binding to DSB initiates resection of DNA ends to various extent. DNA ends with 10–100 nts 3′overhang are recognized and transcribed by RNAPII *in vitro* (72) and may be necessary and sufficient for accumulation of RNAPII at DSBs *in vivo* (27). However, the mechanism that underlays MRN-dependent accumulation of RNAPII at DSBs remains elusive. RNAPII components were also detected as DNA end-binding proteins by mass spectrometry (73). The majority of DSBs in asynchronous cells are repaired by non-homologous end joining (NHEJ), which involves minimal resection by Mre11 3′-5′exonuclease (74,75). Partially resected ssDNA may also stimulate ATR activation during NHEJ in addition to activation of ATM and DNA-PK by non-resected DSBs (76). Given that the expression levels of ATM and DNA-PK are interdependent (77) and that both ATM and DNA-PK activate c-Abl, DSBs may engage RNAPII and c-Abl by a combination of PIKK activities in asynchronous cells.

We postulate that DNA–RNA hybrids form at DSBs as a consequence of pri-DART synthesis and generate dsRNA by transactivation of reverse-oriented transcripts (seDARTs) similarly to the R-loop promoter model, where formation of DNA–RNA-hybrids at RNAPII terminators triggers antisense RNA synthesis *in vivo* (33) and *in vitro* (72). ssDNA, displaced by RNA hybridization, is prone to chemical modification, formation of secondary structures and nicking (78). Interestingly, the endonuclease XPG is involved in resolution of DNA–RNA hybrids at DSBs by cleaving ssDNA (55). Hybrid-prone sequences also strongly correlate with G-quadruplex (G4) predictions at GC-rich loci (79,80). Indeed, recent evidence shows that stabilized G4s increase R-loop levels and cause their spreading (81). Such topological constrains may promote RNAPII recruitment and antisense transcription at DSBs *de novo*. However, inherently unstable transcripts may also be retained on chromatin and contribute to dsRNA formation, since cut *Asi*SI-ER sites are largely associated with protein-coding gene promoters and most of the genome is transcribed pervasively.

The relevance of RNA in the DDR is an emerging concept (21). Findings in *S. cerevisiae* suggest a model of RNA-templated DSB repair, which utilizes exogenous RNA oligonucleotides or endogenous transcripts as templates for DSB repair (82,83). Similarly, nascent RNA forms a complex with actively transcribing RNAPII and some NHEJ factors to mediate DSB repair in HEK293 cells (84). To some extent, however, the developing field remains controversial. Various modes of RNA-dependent DSB repair may coexist, influenced by chromatin and the cell cycle. It has been shown that RNAPII generates damage-induced long non-coding (dilnc)RNA from and toward endogenous DSBs *de novo*, irrespective of the genomic context. To promote DSB repair, dilncRNA may undergo processing into DDRNA and simultaneously represent a substrate for hybridization with mature DDRNA (27). In contrast, others did not detect DDRNA *per se* (56,57). We successfully employed highly sensitive mNET-seq to quantify RNAPII-associated DARTs of low abundance at high resolution. Unlike Michelini *et al.*, our data do not support a uniform *de novo* RNAPII recruitment mechanism, applicable to any DSB, since we detected DARTs at promoter-associated, but not intergenic DSBs, albeit limited to *Asi*SI-ER-restricted loci. While dilncRNA seem to form independent of DNA–RNA hybrids and may partially be retained at DSBs to serve as sensor of lesions, DARTs undergo formation of dsRNA via DNA–RNA intermediates. Interestingly, dilncRNA contributes to HR by recruitment of BRCA1, BRCA2 and Rad51 proteins. In this process dilncRNA pairs with resected DNA strand forming DNA-RNA hybrids, which are recognized by BRCA2 (85). Very recently, nascent RNA synthesis from endogenous DSBs was confirmed (86). Using the I-PpoI-HA endonuclease and deep sequencing, the Visa lab demonstrates accumulation of damage-induced RNA (diRNA) upon cleavage of repetitive rDNA, but not unique genic or intergenic loci. diRNA synthesis involves production of single-stranded precursors, which originate from *de novo* RNAPII recruitment and transcription away from the lesion. Such transcripts either form dsRNA with a preexisting complementary RNA and undergo Dicer processing or mature into diRNA by Dicer-independent trimming. It will be important to determine the regulatory principles and consequences of diRNA synthesis at rDNA lesions.

Collectively, we propose that c-Abl phosphorylates CTD Y1 RNAPII to generate DARTs and form dsRNA. DARTs originate at promoter-associated DSBs and are specifically linked to CTD Y1P. RNA-dependent foci formation is likely connected with canonical DSB repair and in crosstalk with chromatin. Once generated and processed, dsRNA mediate recruitment of 53BP1 and MDC1 to stimulate canonical DSB repair.

## DATA AVAILABILITY

Raw sequencing data are deposited in GEO database (GSE96825). Codes for plots and graphs are provided as Source Code Files 1–5.

## Supplementary Material

Supplementary Data
