# Supplementary material for: Tyrosine kinase c-Abl couples RNA polymerase II transcription to DNA double-strand breaks
Source: Nucleic Acids Res. 2019 Jan 22;47(7):3467–84. doi: 10.1093/nar/gkz024 (PMC6468493; doi:10.1093/nar/gkz024)

SUPPLEMENTARY DATA for

Tyrosine kinase c-Abl couples RNA polymerase II transcription to RNA-dependent recognition of DNA double-strand breaks

Authors: K. Burger[1], M. Schlackow[1] and M. Gullerova[1]*

Affiliations:

[1] Sir William Dunn School of Pathology, University of Oxford, South Parks Road, Oxford, OX1 3RE, UK

*Correspondence to: monika.gullerova@path.ox.ac.uk

Supplementary data contains 12 supplementary figure legends and 2 supplementary tables.

SUPPLEMENTARY FIGURE LEGENDS

Supplementary Figure 1. Analysis of RNAPII in response to DSBs. (**A**) Predicted (top) and cut (bottom) (1) *Asi*SI-ER sites. (**B**) Immunoblots detecting total (8WG16) and phospho-CTD RNAPII levels following incubation with 4-Hydroxytamoxifen (4OHT) for various hours. α-Tubulin, control; II0/IIA, hyper-/hypo-phosphorylated RNAPII; γH2A.X, S139 H2A.X phosphorylation. (**C**) Position-specific quantitation of merged images from Figure 1A using RGB profiler. (**D**) Immunoblots detecting total (8WG16) and phospho-CTD RNAPII upon

preincubation with Flavopiridol (FL), THZ1 or α-Amanitin (α-AM). Rad21, control. (**E**) Imaging and RGB quantitation of CTD Y1P and 53BP1 upon transfection of *Asi*SI-ER-encoding pBABE::*Asi*SI-ER plasmid in mammalian cells. n, number of cells with shown phenotype in %.

Supplementary Figure 2. MRN-dependent formation of CTD Y1P foci at DSBs. (**A**) Imaging and RGB quantitation of CTD Y1P and MRN component Mre11 upon transfection of Mre11-specific siRNA. scrambled siRNA, control. n, number of cells with shown phenotype in %. (**B**) Immunoblots detecting total (8WG16) and phospho-CTD RNAPII levels as well as γH2A.X and MRN-complex components Nbs1 and Mre11 upon transfection of Mre11-specific siRNA. Controls: scrambled siRNA, histone H3. (**C, D**) Imaging of total (N20X) and phospho-CTD RNAPII as well as Mre11 (C) or γH2A.X (D) upon transfection of Mre11-specific siRNA. scrambled siRNA, control. n, number of cells with shown phenotype in %. (**E**) Immunoblots detecting total RNAPII (8WG16) or CTD Y1P levels. Rad21, control. (**F**) Flow cytometry assessing the cell cycle distribution of asynchronous U2OS cells.

Supplementary Figure 3. RNAPII-dependent recruitment of 53BP1 and MDC1 to DSBs and controls for α-AM treatment. (**A**) Imaging of 53BP1 and γH2A.X. Representative images are shown. (**B**) Quantitation of DNA damage recognition, assessing % of cells forming 53BP1 foci. Asterisk, p-value <0.05, two-tailed t-test. Error bar: mean ±SEM, n=3. (**C, D**) as in (A, B) but imaging and quantitation of MDC1. (**E**) RGB quantitation of foci from (C). (**F**) Quantitative PCR (qPCR) of undigested genomic DNA purified from cells using primers specifically targeting across the two *Asi*SI-ER sites acDS-I (cleavage across DS1 site) and acDS-II as described in (2) a non-restricted control locus (no DSB) and exons within two

housekeeping genes (*GAPDH*, *ACTB*). Induction of DSBs is shown in %. Values were normalised to signals measured for *ACTB* (left) or *GAPDH* (right) and calculated using the ΔΔcT method. % DSB values in absence of 4OHT were set to 0. Asterisk, p-value <0.05, two-tailed t-test. Error bar: mean ±SEM, n=3. (**G**) Immunoblots detecting Ki-67, MDC1, 53BP1 and HA-tagged *Asi*SI-ER. Histone H3, control. (**H**) Flow cytometry analysis assessing the cell cycle distribution of asynchronous U2OS cells. n, number of foci per cell.

Supplementary Figure 4. PIKK-dependent formation of CTD Y1P foci at DSBs. (**A**) Imaging of CTD Y1P and γH2A.X. ATMi, ATM inhibitor KU-55933; ATRi, ATR inhibitor VE-821; PIKKi, PIKK inhibitor LY294002. n, number of cells with shown phenotype in %. (**B**) Imaging of CTD Y1P and 53BP1 upon transfection of scrambled siRNA (siControl) or siRNA targeting ATM, ATR or DNA-PKcs. n, number of cells with shown phenotype in %. (**C**) as in (B) with larger field representative images shown. (**D**) Quantitation of CTD Y1P and γH2A.X from selected images from (A, B). (**E**) Immunoblots detecting ATM, ATR, DNA-PKcs, 53BP1 and CTD Y1P upon transfection with specific siRNA. siPIKKs mix, equimolar mixture of PIKK-targeting siRNAs; scrambled siRNA, control. Histone H3, control.

Supplementary Figure 5. c-Abl-dependent formation of CTD Y1P foci at DSBs. (**A, B**) Imaging of CTD Y1P, γH2A.X and total RNAPII (N20X) upon (A) γ-irradiation or (B) incubation with 4OHT. Representative images are shown. (**C, D**) Immunoblots detecting total RNAPII (8WG16), CTD Y1P and γH2A.X upon (C) increasing doses of γ-irradiation or (D) various time points after γ-irradiation. β-Tubulin, control. (**E**) Imaging of CTD Y1P, γH2A.X and p-c-Abl upon increasing doses of γ-irradiation. Representative images are shown. (**F**)

Immunoblots detecting CTD Y1P, p-c-Abl and total c-Abl upon immunoprecipitation. IP controls: HA, Grp75; loading controls: immunoglobulin chains (IgGs); asterisk, unspecific.

Supplementary Figure 6. c-Abl catalyses formation of CTD Y1P foci at DSBs. (**A**) Immunoblots detecting total RNAPII (8WG16), CTD Y1P, and c-Abl upon transfection with siRNA specific for c-Abl. (**B, C**) Imaging of CTD Y1P, p-c-Abl and γH2A.X upon transfection with siRNA specific for c-Abl. Scrambled siRNA, control. Representative images are shown. Broken circle, c-Abl-depleted nucleus. (**D, E**) Immunoblots detecting total RNAPII (8WG16), CTD Y1P, and c-Abl upon transfection with siRNA specific for c-Abl untranslated region (UTR) (left panel) or upon cotransfection of untranslated region-specific c-Abl siRNA (siAbl-UTR) with enhanced GFP-tagged c-Abl kinase active/kinase dead constructs (eGFP-Abl KA/KD) or copepod GFP expressing vector (CopGFP). Controls: scrambled siRNA, Ponceau S, Histone H3, β-Tubulin. Asterisk, unspecific. Note that CopGFP is not recognised by the GFP antibody.

Supplementary Figure 7. Analysis of RNA levels in response to DSBs. (**A**) Quantitative real-time PCR (qRT-PCR) of reverse-transcribed total RNA, assessing RNA levels originating upstream of DS1 using site-specific primers. Ratios of signals in presence of 4OHT normalised to signals in absence of 4OHT are shown. Control loci: *HPRT1*, no DSB. Asterisk, p-value <0.05, two-tailed t-test. Error bar: mean ±SEM, n=3. (**B**) qRT-PCR of nascent, 4-thiouridine-(4sU)-tagged RNA, assessing RNA levels upstream of DS1 and control loci. Ratios of signals in presence of 4OHT are normalised to signals in absence of 4OHT. Asterisk, p-value <0.05, two-tailed t-test. Error bar: mean ±SEM, n=3. (**C**) Ethidium bromide-stained agarose gels of total, unlabeled and 4sU-tagged RNA (top) and immunoblots detecting γH2A.X (bottom). 28S/18S, ribosomal RNA and α-Tubulin, controls. (**D**)

Autoradiograph detecting end-labeled transcripts, upon immunoprecipitation with phospho-CTD antibodies (mNET-IP) and PAGE separation. RNAPII-associated transcripts were quantified as relative signal sum. Values in absence of 4OHT were set to 1. (E) Immunoblots detecting total RNAPII (8WG16) and CTD Y1P in mNET-IP samples immunoselected using 3D12 antibody; GFP, control. (**F**) Autoradiograph of RNAPII CTD Y1P-associated transcripts. GFP, control. (**G**) mNET-seq depicting average sense/antisense signals across 94 genic non-restricted *Asi*SI-ER sites. Left/right, CTD Y1P/total RNAPII (8WG16) IP; dashed line, *Asi*SI site. (**H**) as in (G) but showing 94 cut, genic (promoter, exons, introns) *Asi*SI-ER sites (left panels) and 94 uncut, genic (promoter, exons, introns) control sites (right panels) in both wild type U2OS (wt) and *Asi*SI-ER U2OS (rec.) cells in presence of 4OHT. Left/right panels, CTD Y1P/total (8WG16) RNAPII IP. All profiles are plotted around *Asi*SI-ER sites (dashed line) +/-2kb.

Supplementary Figure 8. mNET-seq traces at selected genes. Genome browser snapshots showing mNET-seq signals associated with total (8WG16) or CTD Y1P RNAPII on selected genic regions in presence of 4OHT in wild type (wt) U2OS cells and *Asi*SI-ER U2OS (rec.) cells (bottom). Blue/red lines, sense/antisense transcripts. *Asi*SI-ER sites are positioned in the centre of each graph. Green boxes, regions of DART synthesis. Samples are labeled on the left side. Positions of loci are depicted on the top of each graph.

Supplementary Figure 9. mNET-seq traces at intergenic regions. Genome browser snapshots showing mNET-seq signals associated with total (8WG16) or CTD Y1P RNAPII on selected intergenic regions in presence of 4OHT in wild type (wt) U2OS cells and *Asi*SI-ER U2OS (rec.) cells: Blue/red lines, sense/antisense transcripts. *Asi*SI sites are positioned in the centre of each graph. Samples are labeled on the left side.

Supplementary Figure 10. Imaging of RNA in response to DSBs. (**A**) Assessment of RNA at DS1. An equimolar mixture of 18 primary DNA oligonucleotide probes, which are locus-specific for a region up to 500 nts distant from DS1 (probe mix DS1) was used. White box, 5x zoom. Representative images are shown. (**B**) RGB quantitation of RNA FISH from Figures 2C (left) and 2D (right).

Supplementary Figure 11. Controls for assessment of damage-induced RNA. (**A**) Imaging and RGB quantitation of CTD Y1P and DNA-RNA hybrids (S9.6). Representative images are shown. (**B**) Immunoblots detecting total Dicer (A-2), γH2A.X, total RNAPII (8WG16) and CTD Y1P following transfection of scrambled (shControl) or Dicer-targeting (shDicer) short-hairpin RNA. Histone H3, control. (**C**) qRT-PCR of cDNA after RNA IP with dsRNA antibody J2 (J2 RIP) and reverse transcription with forward (fwd)- and reverse (rev)-oriented primers, specifically recognising a region up to 1000 nts distant from DS1 in presence of 4OHT (J2+). Values for J2 RIP at primer site DS1+80 were set to 1 for both forward- and reverse-oriented cDNA sets. Asterisk, p-value <0.05, two-tailed t-test. Error bar: mean ±SEM, n=3. (**D**) *In vitro* analysis of dsRNA upon expression of Dicer-specific short-hairpin (sh)RNA and J2 RIP. Equal amounts of total RNA inputs (IN, control) or immunoselected RNA (IP) were incubated with bovine serum albumin (BSA) (-) or RNaseIII (+) and stained with SYBR gold upon gel separation. AU, arbitrary units.

Supplementary Figure 12. DNA-RNA hybrid-dependent recruitment of 53BP1 and MDC1 to DSBs and onset of DSB signaling. (**A**) As in Figure 5, but using RNA immuno-depleted with beads (mock IP), S9.6, J2 or ssDNA specific antibodies for add back. Representative images are shown (top). Quantification of cells with foci staining double-positive for CTD

Y1P and 53BP1 (bottom). The experiment was done in triplicates. 60 cells were counted per replicate. Cells were scored based on average number n<10 of Y1P/53BP1 double foci (yellow) per cell. Asterisk, p-value <0.05, one-tailed t-test. Error bar: mean ±SEM, n=3. (**B**) Immunoblots detecting HA-53BP1 and GFP-RNaseH1 following transient transfection of *pHAGE-N-FLAG-HA-53BP1* plasmid, expressing HA-tagged, full length 53BP1 or transfection of pEGFP-M27, expression GFP-tagged RNaseH1, or co-transfection of both plasmids. Mock, non-transfected cells; Ponceau S, control. (**C**) Immunoblots detecting GFP-RNaseH1 transfected with pEGFP-M27 plasmid after fluorescence-activated cell sorting (FACS). Rad21, control. (**D-H**) Quantitation of phospho-ATM (D), γH2A.X (E), phospho-Chk1 (F), total ATM (G), and total p53 (H) levels. Signals after 2 hours 4OHT incubation without chase (2h+0h) are set as 1. Asterisk, p-value <0.05, two-tailed t-test. Error bar: mean ±SEM, n=3. (**I**) Immunoblots detecting phospho-ATM, GFP-RNaseH1 and γH2A.X levels in non-sorted cells. Ponceau S, control.

SUPPLEMENTARY TABLES

**Primer**	**Sequence (5'-3')**
ACTB-fwd	ATGTTTGAGACCTTCAACACC
ACTB-rev	ATCTTCATGAGGTAGTCAGTCAG
ac DS-I-fwd	GATGTGGCCAGGGATTGG
ac DS-I-rev	CACTCAAGCCCAACCCGT
ac DS-II-fwd	GAGGAGCCTCTCCTGCAGC
ac DS-II-rev	GAACCAGACCTACCTCCAGGG
GAPDH-fwd	AACCTGCCAAATATGATGAC
GAPDH-rev	AGGAAATGAGCTTGACAAAG

DS2-fwd	TGCCGGTCTCCTAGAAGTTG
DS2-rev	GCGCTTGATTTCCCTGAGT
DS1-fwd	GATTGGCTATGGGTGTGGAC
DS1-rev	CATCCTTGCAAACCAGTCCT
300-fwd	AGGACTGGTTTGCAAGGATG
300-rev	ACCCCCATCTCAAATGACAA
500-fwd	CCTGGATATGAGTTTGATCAGC
500-rev	CTCTCCTTTCGCTGACACTG
1000-fwd	AGGAATTGACTGCGGTGTTC
1000-rev	GGGGAGGAGGAAAGGTGTAG
2000-fwd	GCCATAACAGAGGGTGGAAA
2000-rev	AACTTTAGGATGGGGCTGCT
HPRT1-fwd	AGATGTGATGAAGGAGATGG
HPRT1-rev	AATAGCTCTTCAGTCTGATAAAATC
335-fwd	GAATCGGATGTATGCGACTGATC
335-rev	TTCCAAAGTTATTCCAACCCGAT
no DSB-fwd	ATTGGGTATCTGCGTCTAGTGAGG
no DSB-rev	GACTCAATTACATCCCTGCAGCT

Supplementary Table 1. Primer pairs used for ChIP and qRT-PCR.

**Primer**	**Sequence (5'-3')**
DS1-fwd-1 (Chr. 1)	TCCTCACTCAAGCCCAACCCGTGGCCCCCACCCCTTCCCT CCTCCATTTCCATCCATTCTGGCACTCTTGAAAAAGTCATG CTGTTTC

DS1-fwd-2 (Chr. 1)	CCCCACTAACTCGAGCCCGCACCTACGCATGCCTGAACCA GCATGTGATCCCGGCCGACTTGGCACTCTTGAAAAAGTCA TGCTGTTTC
DS1-fwd-3 (Chr. 1)	CCTTTTCACACTCTCTCTAGCCCTGCCCTCTCTCAGGCACC TTCCAAAGTTATTCCAACCCTGGCACTCTTGAAAAAGTCA TGCTGTTTC
DS1-fwd-4 (Chr. 1)	GTCATTTTTCTTCCTTCTTCAGTCTCACATGTCCCTTTCATT CTTAGTTTGCTGATCAAACTGGCACTCTTGAAAAAGTCAT GCTGTTTC
DS1-fwd-5 (Chr. 1)	TTTTGTTCCCTCCTTTAGAACTGAATAACAATGCTAACGCC TCTCCCACTCCCTCTAGCCTTGGCACTCTTGAAAAAGTCAT GCTGTTTC
DS1-fwd-6 (Chr. 1)	CTGAGTCACACTCCACAGCCAATCCCAGGCCACATCCAGG CTCGCCCCTACCTGTACACAATGGCACTCTTGAAAAAGTC ATGCTGTTTC
DS1-fwd-7 (Chr. 1)	CCTACACCTTTCCTCCTCCCCCAGCTAATATGATTGTTGCT TCTTTGCCGATTACAGCTTTTGGCACTCTTGAAAAAGTCAT GCTGTTTC
DS1-fwd-8 (Chr. 1)	AGTGAGACAACAAACAAGATCACACTATTTTTGCCTAACC AGATTGGCACTCTTGAAAAAGTCATGCTGTTTC
DS1-fwd-9 (Chr. 1)	AAAAGAAAAACAGCCCTTGGTGTCACGAACAGAACTGAC ACTTTAGTGTTGTTAGGAATTGTGGCACTCTTGAAAAAGT CATGCTGTTTC
DS1-fwd-10 (Chr. 1)	CTTTCATAGCACTTACCACCCTCTAAAGTCATTTTTTATAC TTACTTGTCTATTGCTGGCACTCTTGAAAAAGTCATGCTGT

	TTC
DS1-fwd-11 (Chr. 1)	AACTCCTATTTCCCTTTCCAGAATCATTTTTGCAAGGCTGCTTCCTTACCATTCAATTCTGATGGCACTCTTGAAAAAGTCATGCTGTTTC
DS1-fwd-12 (Chr. 1)	CTCCTGACCTGATGATCCACCCACCCACCTTGGCCTCCCAAAGTGCTGGGATTACAGGCTGGCACTCTTGAAAAAGTCATGCTGTTTC
DS1-fwd-13 (Chr. 1)	GCAGTTCTCCTGCCTCAGTCTCCTGAGTAGCTAGGATTACAGGCATGCACCACCTGGCACTCTTGAAAAAGTCATGCTGTTTC
DS1-fwd-14 (Chr. 1)	AAGATAAAAGGGTAAAGGGCAATATACCTATTAGCTAGTAATTTCTGGCACTCTTGAAAAAGTCATGCTGTTTC
DS1-fwd-15 (Chr. 1)	GCAAGGAAAAAGGCCTAATCTACATAATTTTTATTCAGAAAACAAGGGTATAAGGGTATGGCACTCTTGAAAAAGTCATGCTGTTTC
DS1-fwd-16 (Chr. 1)	AGAAATGAAATAACTTGCCCAAGGTAACACAGCTAGAAAATCGCAGAGGTGGAATTTGTGGCACTCTTGAAAAAGTCATGCTGTTTC
DS1-fwd-17 (Chr. 1)	GTACTCTCCTAAATATTGTTGGTTTTGTTTTGGTTGATTGATTCATTAAATCATTTTGGCACTCTTGAAAAAGTCATGCTGTTTC
DS1-fwd-18 (Chr. 1)	CTATAGATTTAAAAACAGATTGTGACCCAAAAAAAGTTTGGCACTCTTGAAAAAGTCATGCTGTTTC
no DSB-1 (Chr. 22)	GAGGCTGAAACACAGAGATCATATGTCTGAGAAATAAGG

	AAATTCCATACTAATGTGGCACTCTTGAAAAAGTCATGCTGTTTC
no DSB-2 (Chr. 22)	CAAAGGTGTGTTGTCAAAAGCAGAGAAAGTGAGACTTTCCATTGTGGTTTAAATGGCACTCTTGAAAAAGTCATGCTGTTTC
no DSB-3 (Chr. 22)	GTGGCTTAGCTCCTATTCACCCTCCTCACGCAGGCACAGGTAAGACTCACTGGCACTCTTGAAAAAGTCATGCTGTTTC
no DSB-4 (Chr. 22)	CTCCCCCCCATGTCTCAGAAGCTGTTTTCAGGCAAGTCAGCCAGGGAGGAAAGTGGTGGCACTCTTGAAAAAGTCATGCTGTTTC
no DSB-5 (Chr. 22)	TCCTTGAGAGGAAATTCGGGCACTGTACGGGAGGAAAACACAGGACAGGGAGGCCTCTTGGCACTCTTGAAAAAGTCATGCTGTTTC
Secondary probe	488-GAAACAGCATGACTTTTTCAAGAGTGCCA

Supplementary Table 2. DNA oligonucleotides used for sm RNA FISH.

References

1. Aymard, F., Bugler, B., Schmidt, C.K., Guillou, E., Caron, P., Briois, S., Iacovoni, J.S., Daburon, V., Miller, K.M., Jackson, S.P. *et al.* (2014) Transcriptionally active chromatin recruits homologous recombination at DNA double-strand breaks. *Nature structural & molecular biology*, **21**, 366-374.
2. Zhou, Y., Caron, P., Legube, G. and Paull, T.T. (2014) Quantitation of DNA double-strand break resection intermediates in human cells. *Nucleic acids research*, **42**, e19.

Supplementary Figure 1

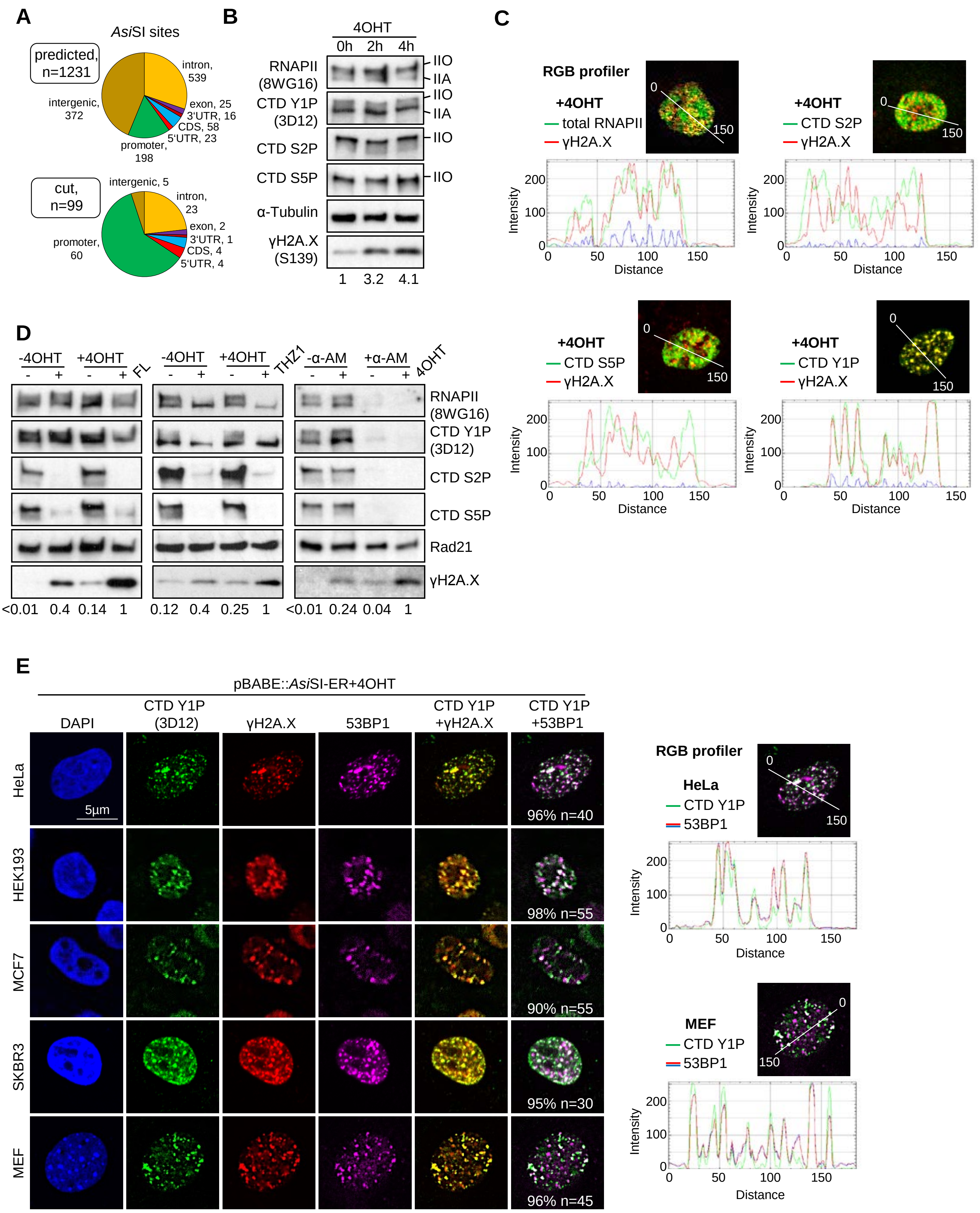

Supplementary Figure 2

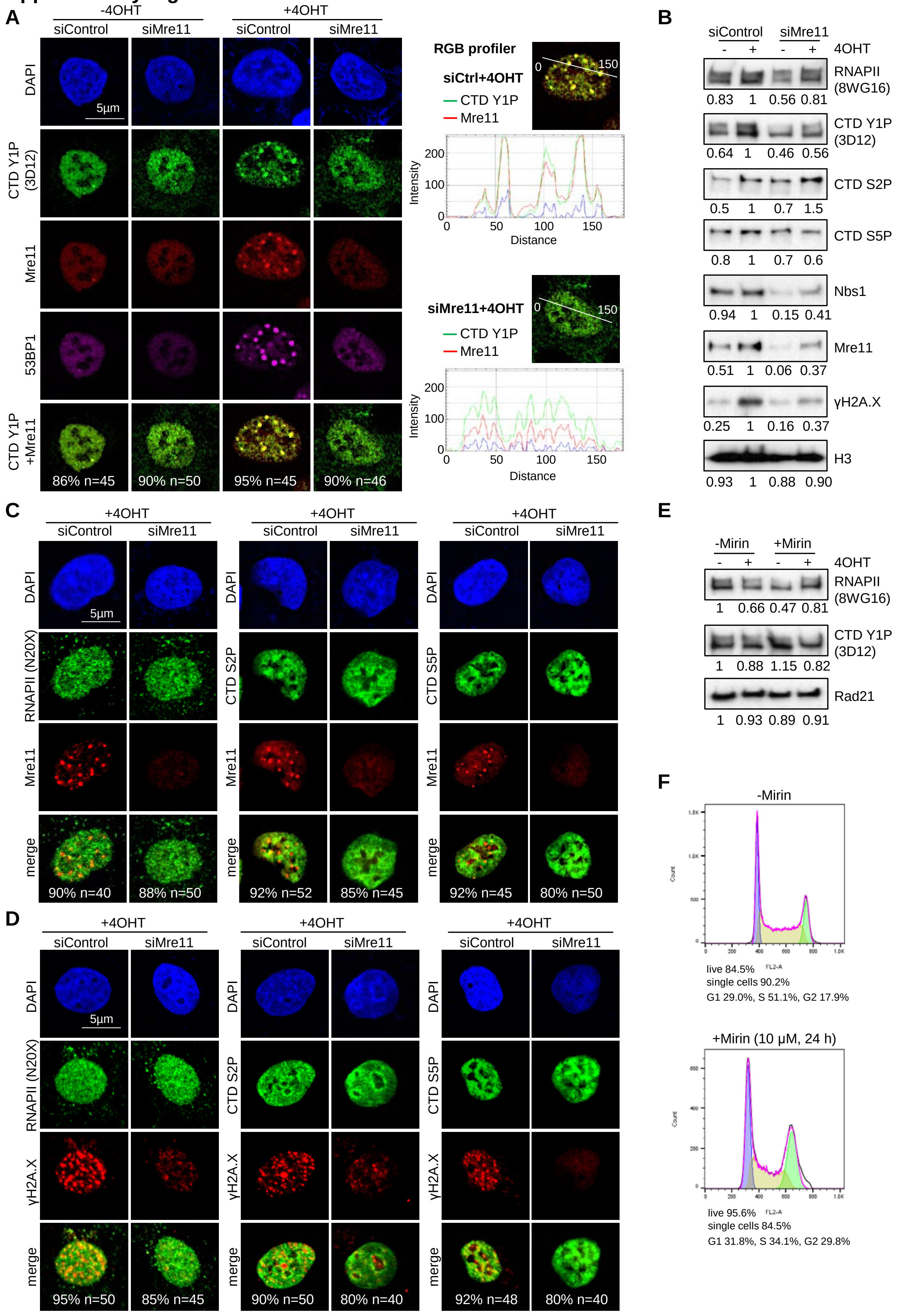

Supplementary Figure 3

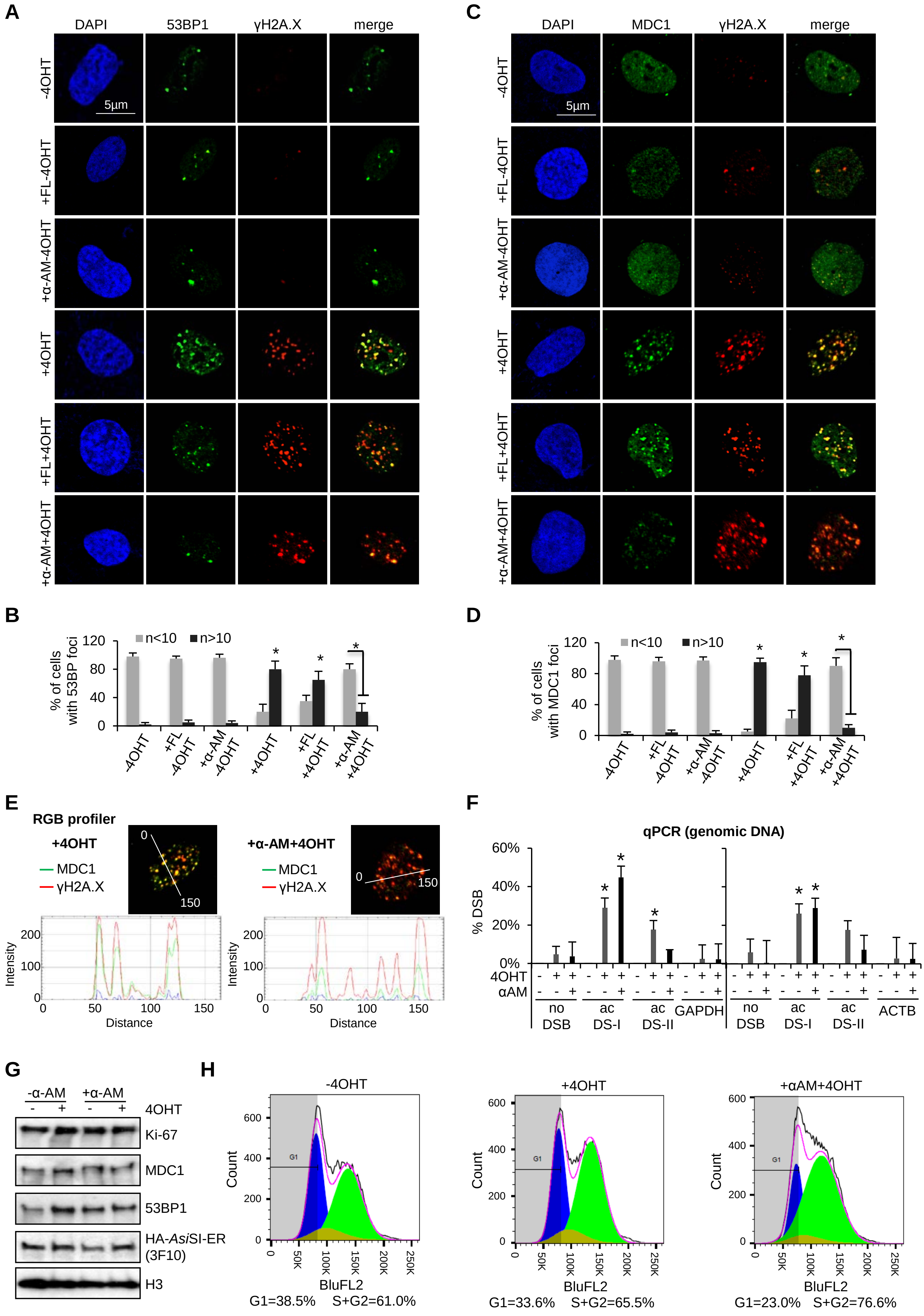

Supplementary Figure 4

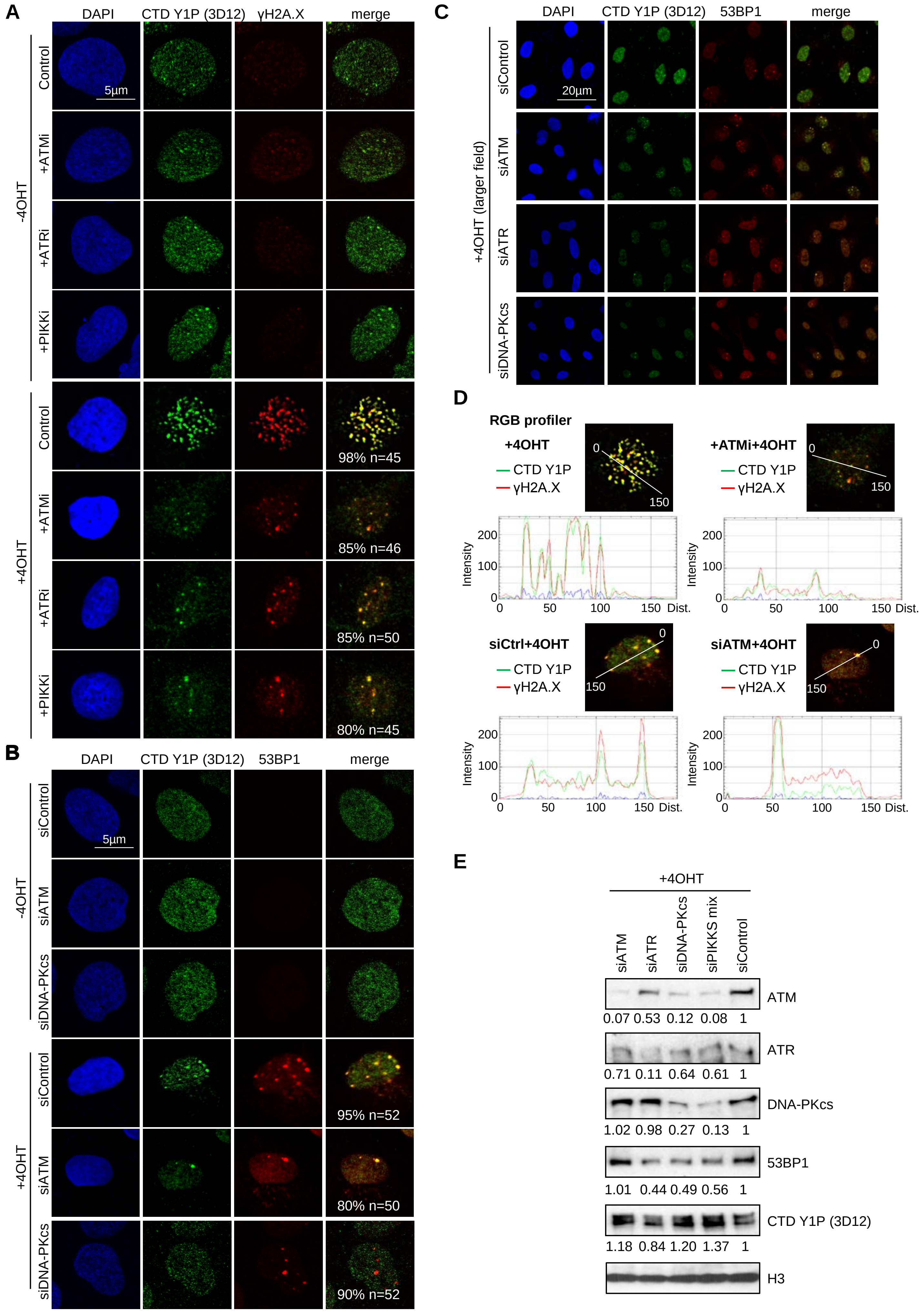

Supplementary Figure 5

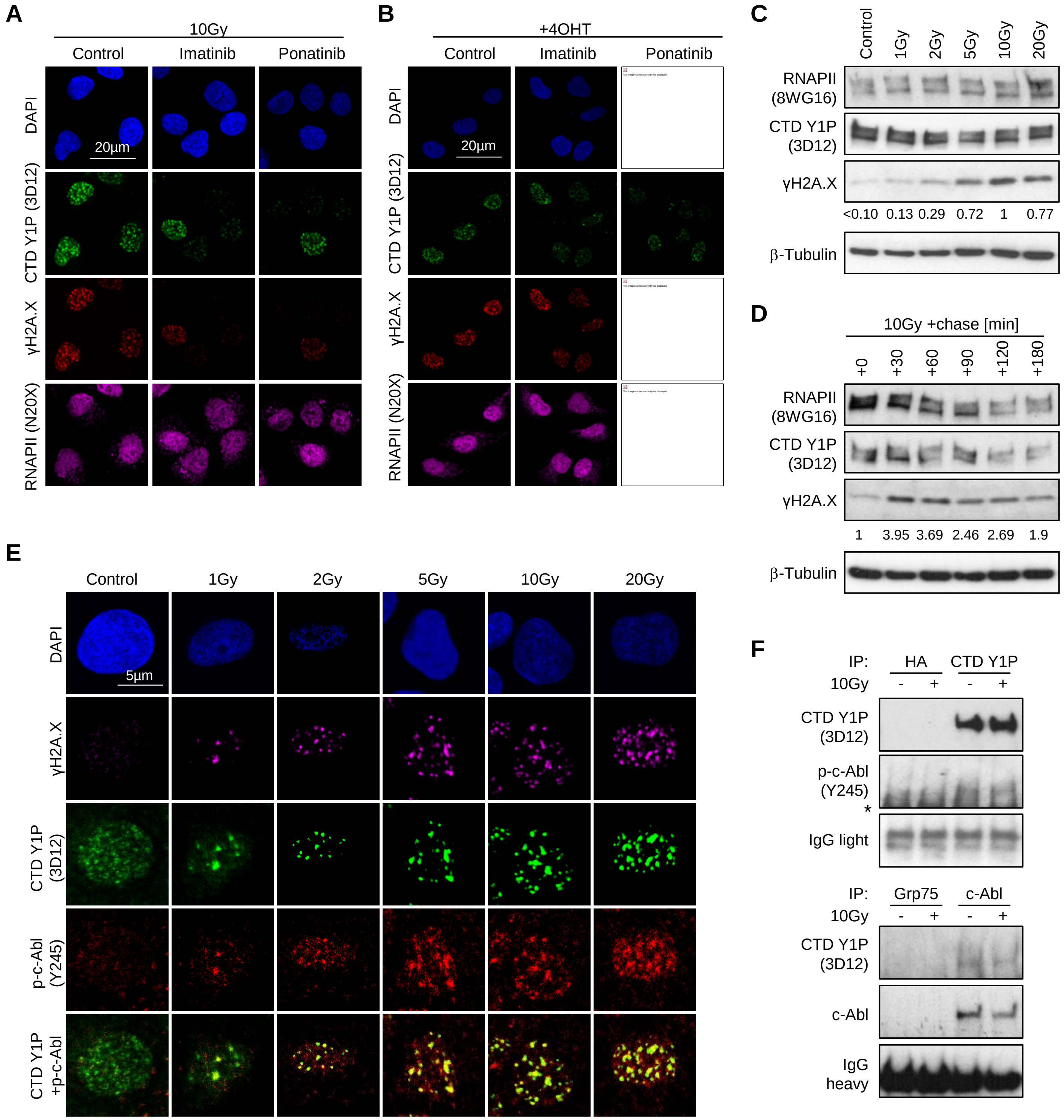

Supplementary Figure 6

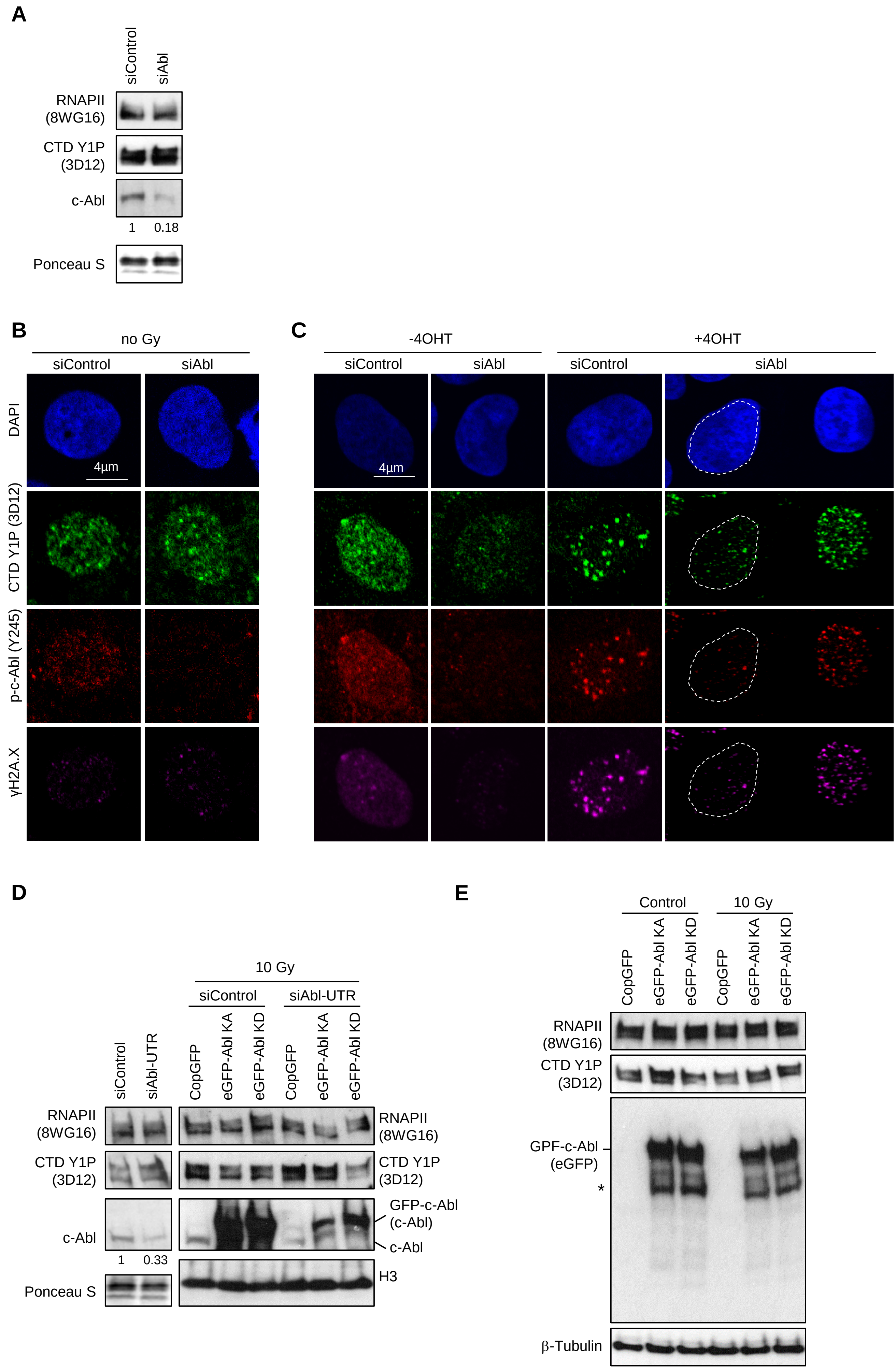

Supplementary Figure 7

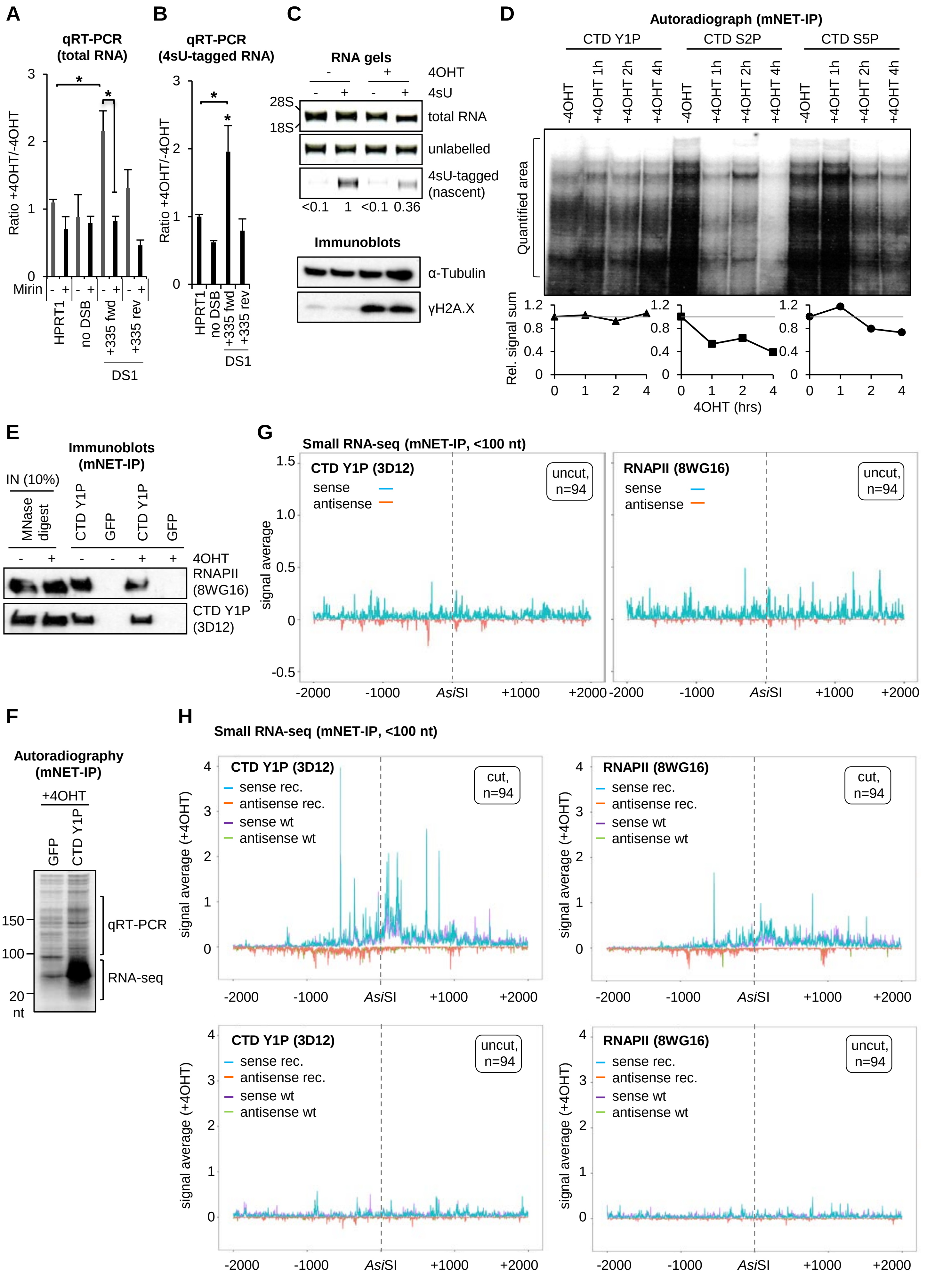

Supplementary Figure 8

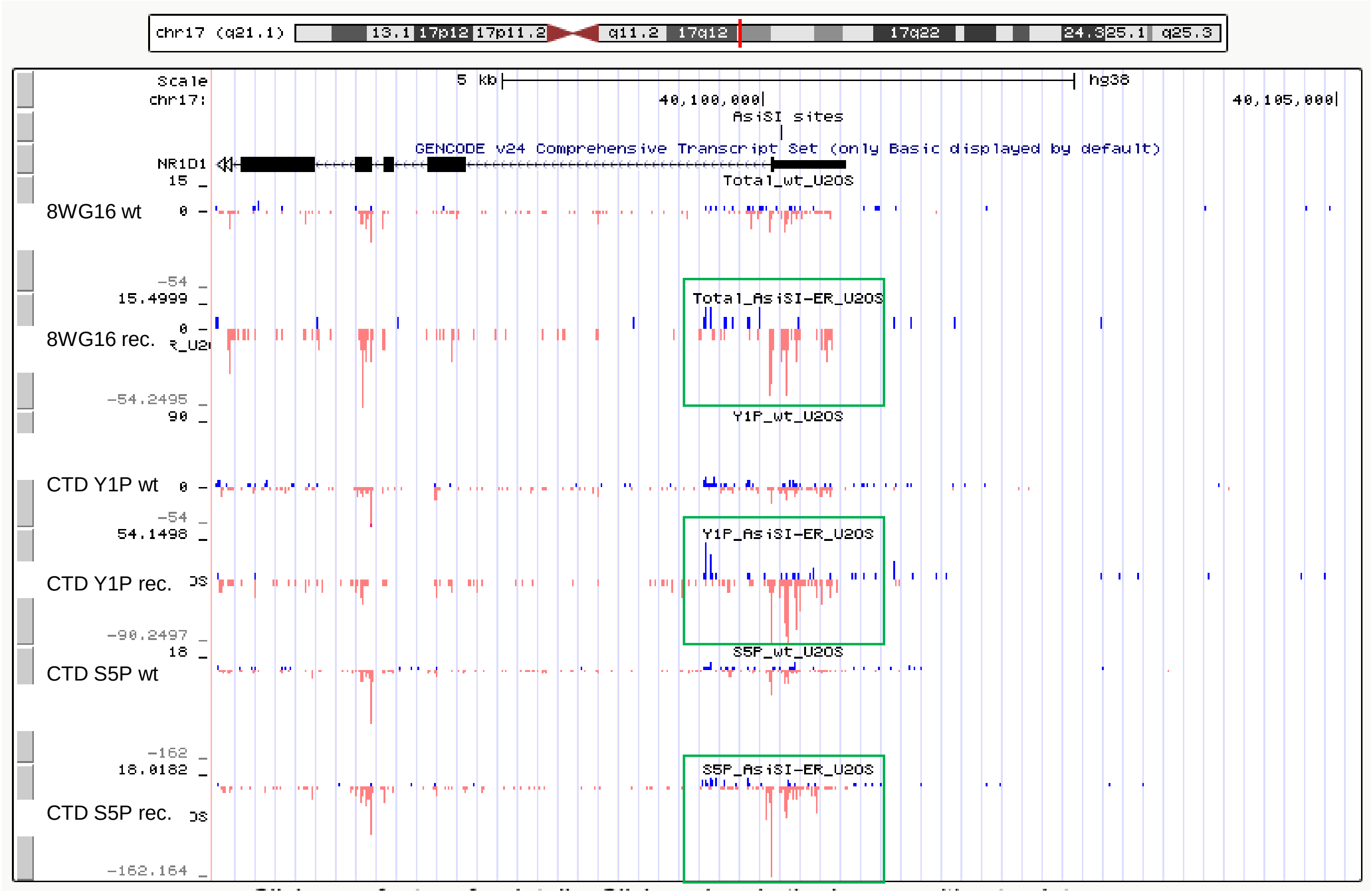

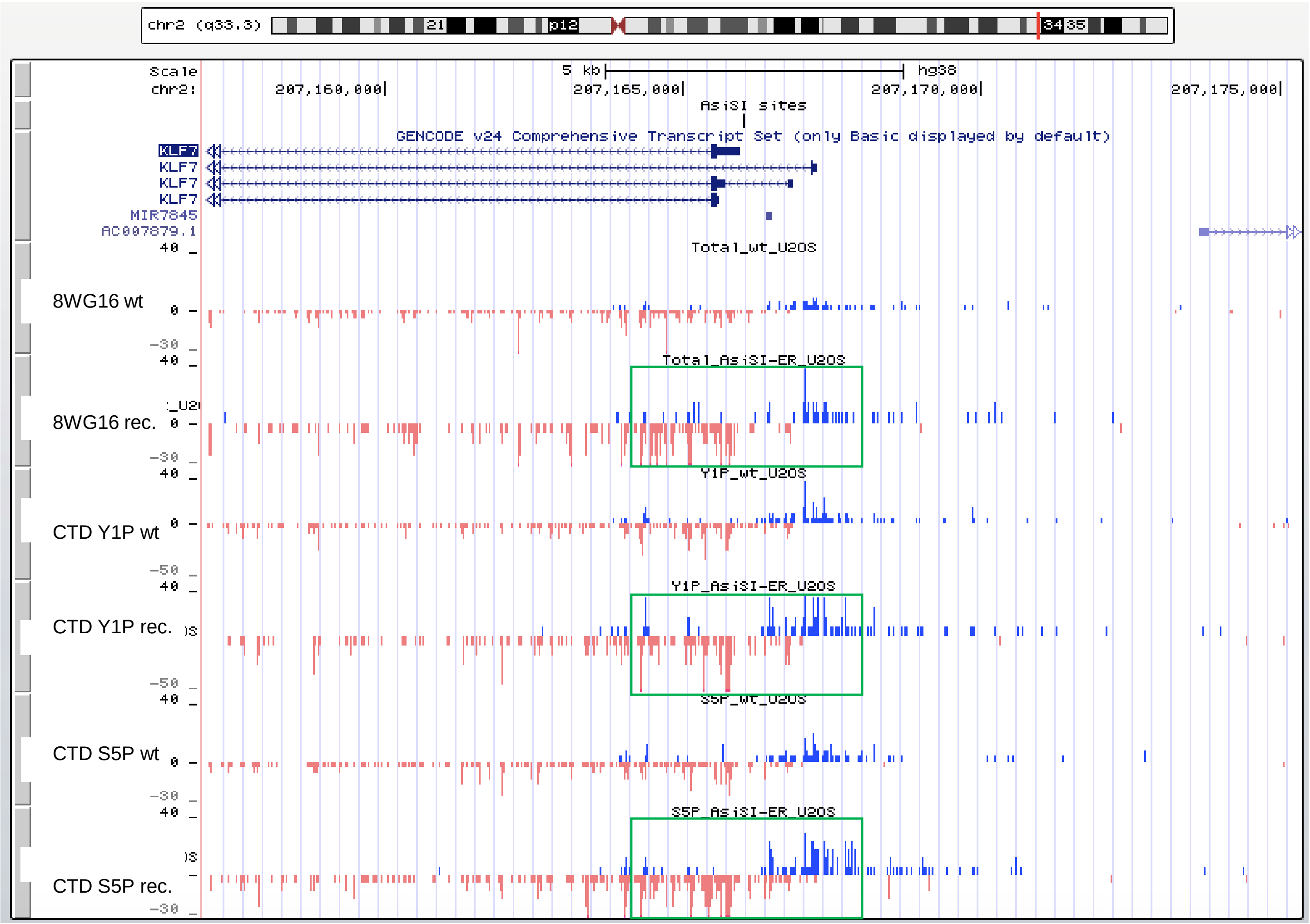

Supplementary Figure 9

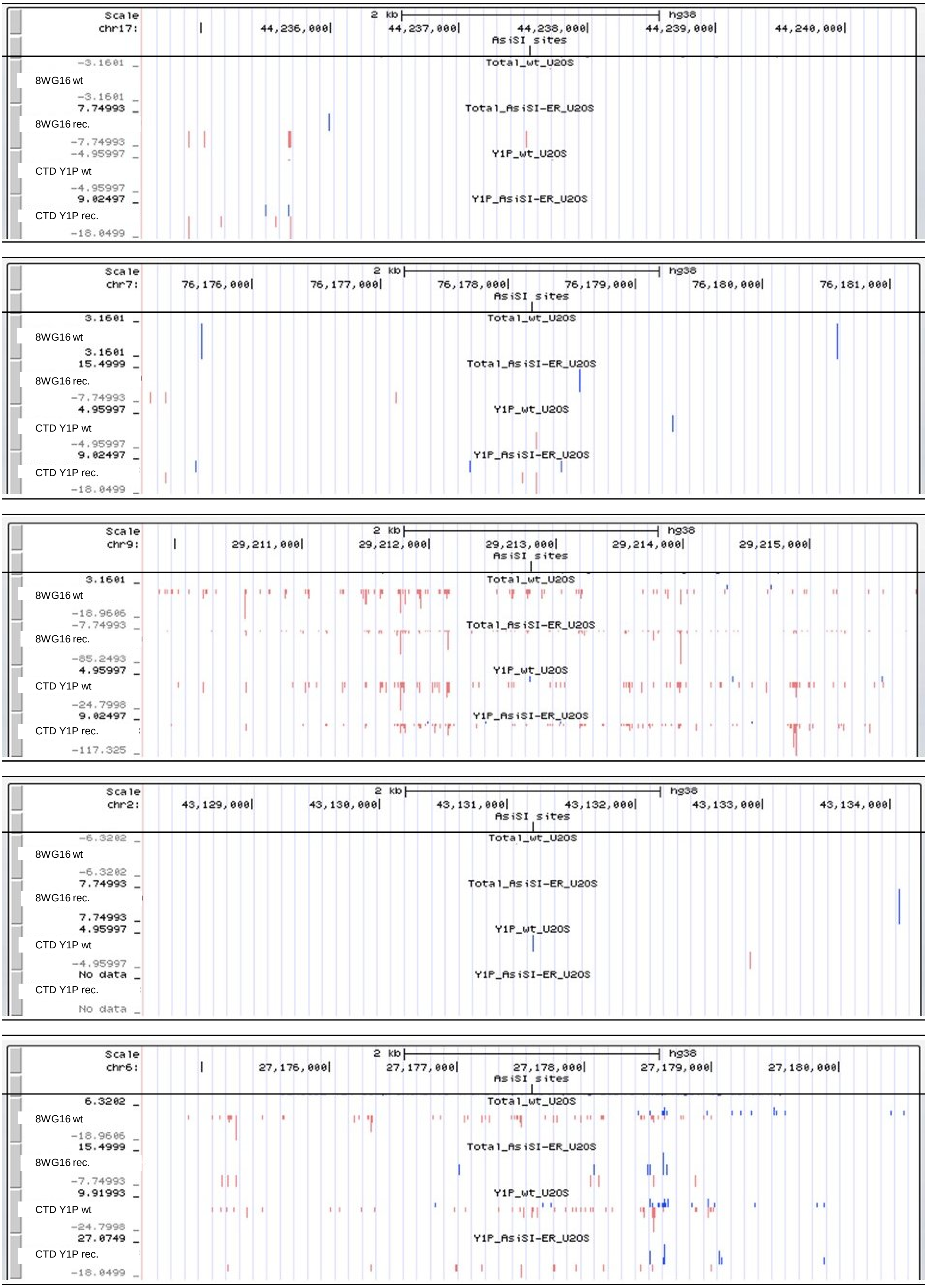

Supplementary Figure 10

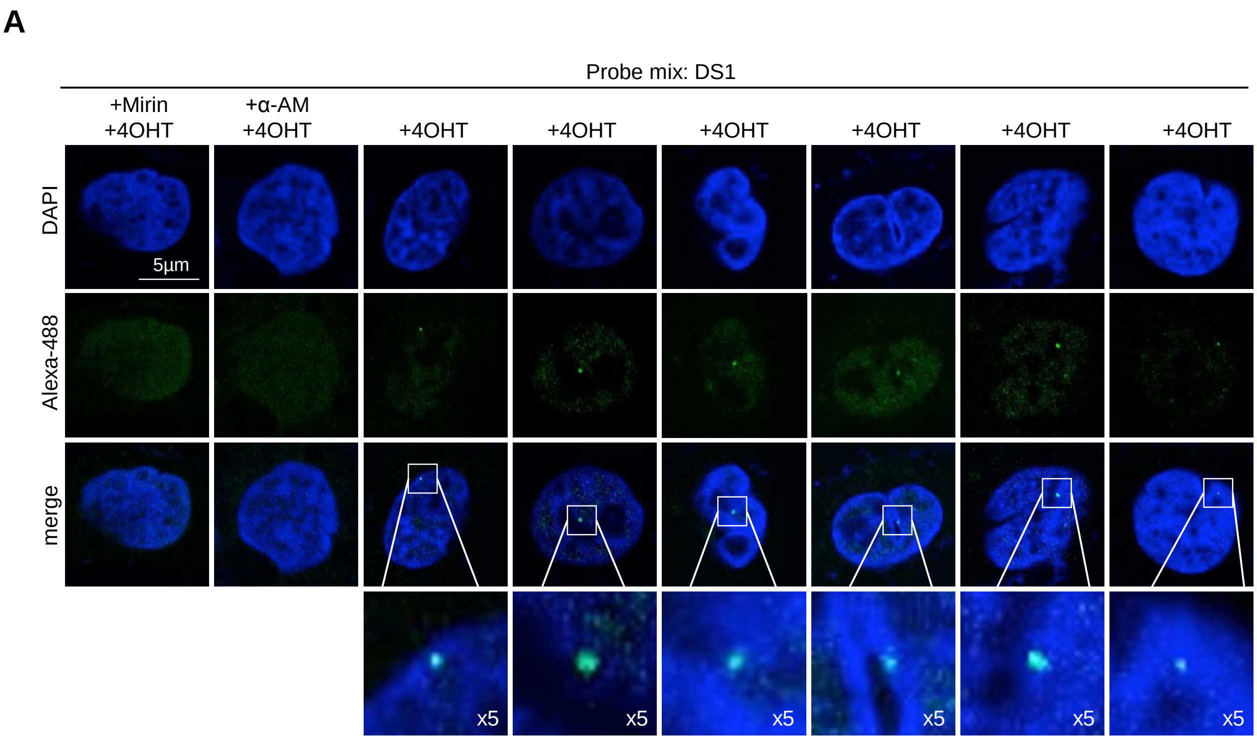

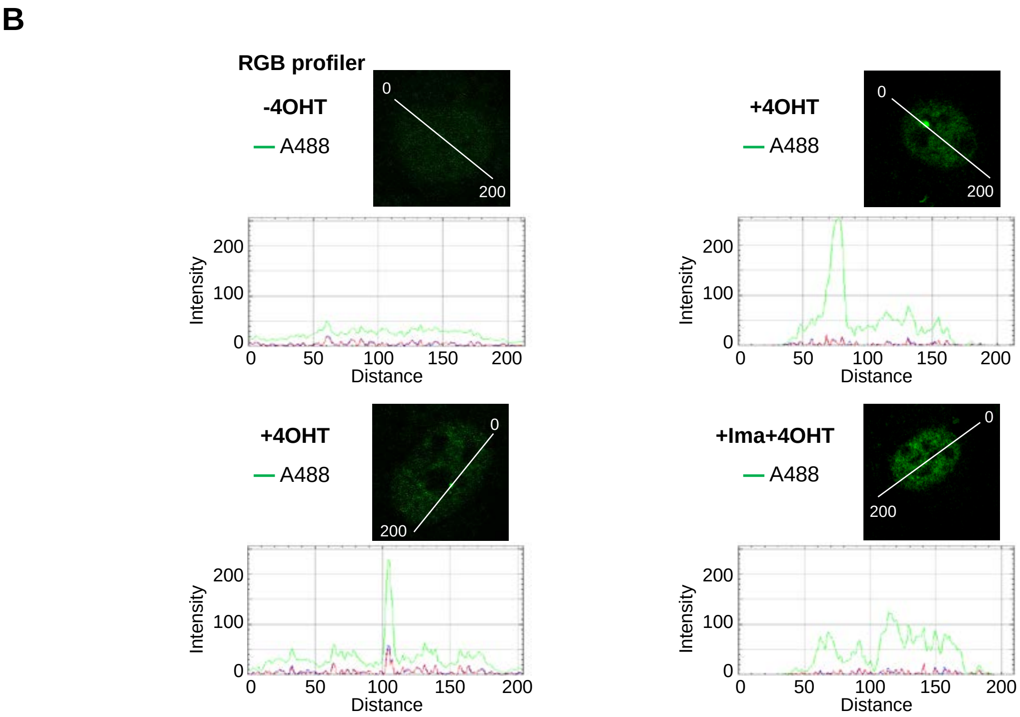

Supplementary Figure 11

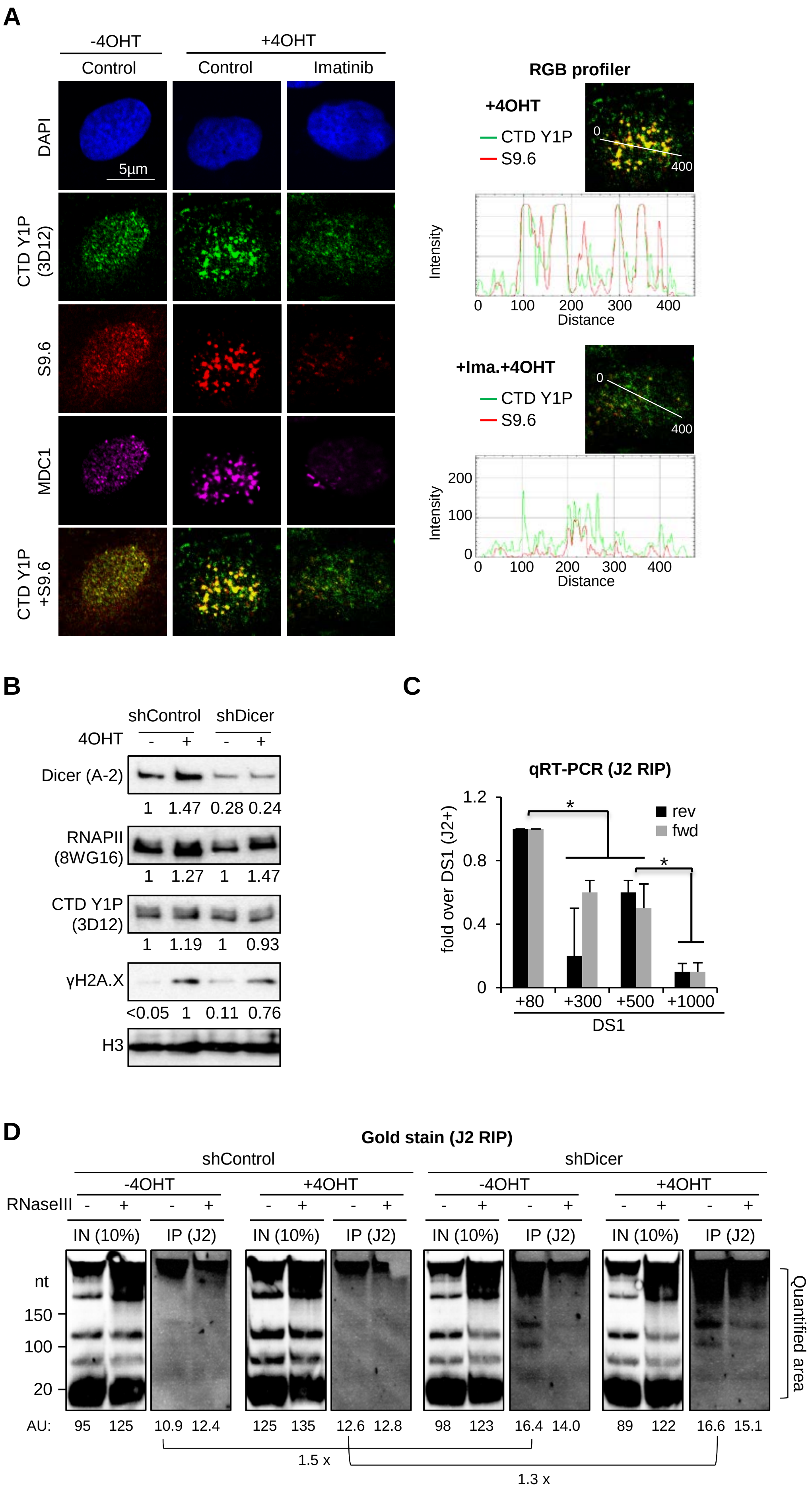

Supplementary Figure 12

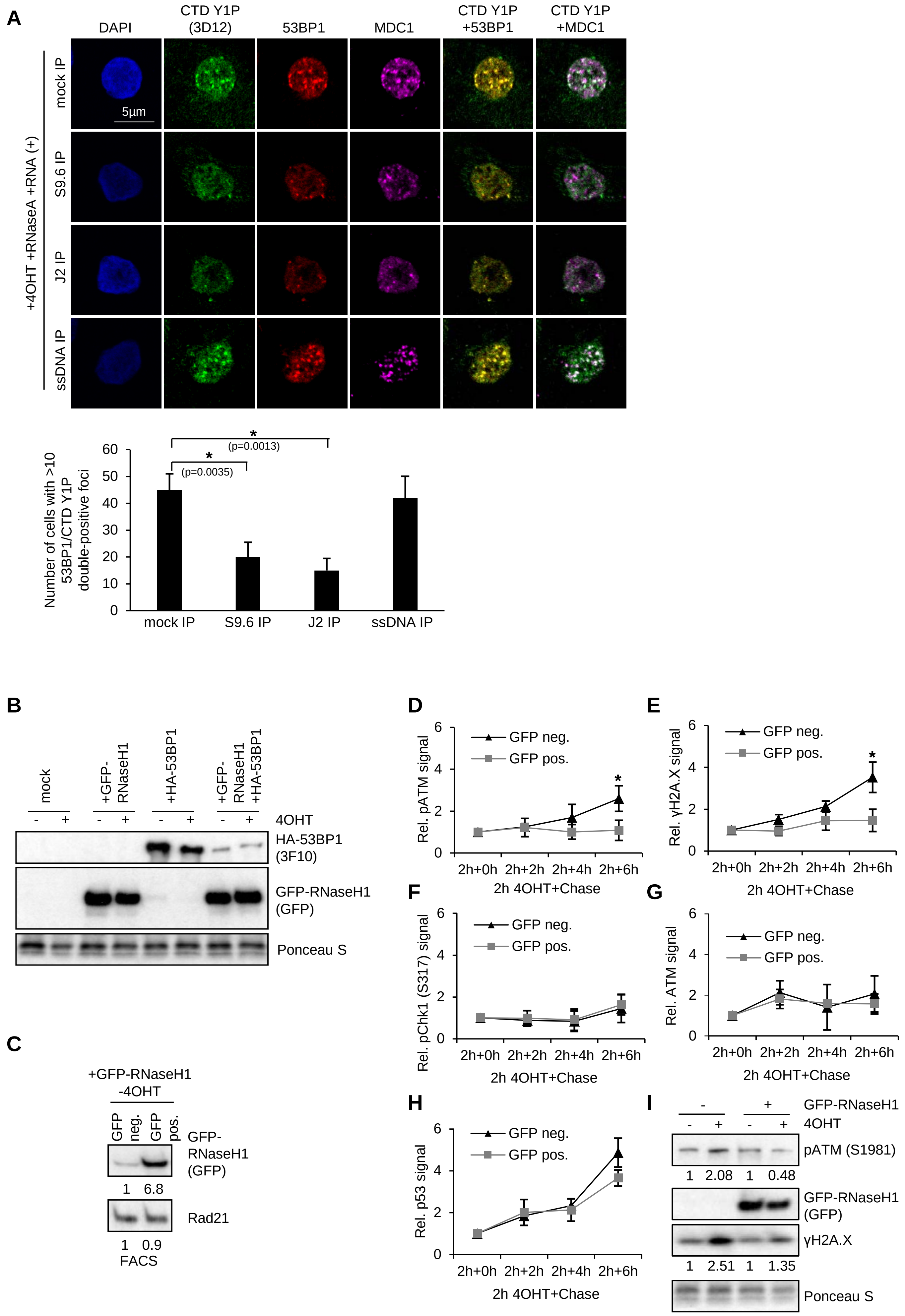

Supplement: Supplementary Data [file gkz024_supplemental_files.pdf]
